# Optical soliton perturbation with complex ginzburg–landau equation having multiplicative white noise and nine forms of self–phase modulation structures^☆^

**DOI:** 10.1016/j.mex.2025.103544

**Published:** 2025-07-30

**Authors:** Elsayed M.E. Zayed, Basel M.M. Saad, Ahmed H. Arnous, Yakup Yildirim, Ibrahim Zeghaiton Chaloob, Ahmed Shaker Mahmood, Luminita Moraru, Hamlet Isakhanli, Anjan Biswas

**Affiliations:** a Department of Mathematics, Faculty of Science, Zagazig University, Zagazig 44519, Egypt; b Department of Mathematics, Faculty of Science, Arish University, North Sinai 45511, Egypt; c Department of Engineering Mathematics and Physics, Higher Institute of Engineering, El Shorouk Academy, Cairo 11837, Egypt; d Department of Computer Engineering, Biruni University, Istanbul 34010, Turkey; e Mathematics Research Center, Near East University 99138 Nicosia, Cyprus; f Al-Esraa University, College of Administration and Economics, Department of Business Administration, Baghdad 10069, Iraq; g Al-Nibras University, Tikrit, 34001, Iraq; h Faculty of Sciences and Environment, Department of Chemistry, Physics and Environment, Dunarea de Jos University of Galati, 47 Domneasca Street 800008, Romania; i Department of Physics, Sefako Makgatho Health Sciences University, Medunsa 0204, South Africa; j Department of Mathematics, Khazar University, Baku, AZ 1096, Azerbaijan; k Department of Mathematics and Physics, Grambling State University, Grambling, LA 71245–2715, USA; l Department of Physics and Electronics, Khazar University, Baku, AZ 1096, Azerbaijan; m Department of Applied Sciences, Cross–Border Faculty of Humanities, Economics and Engineering, Dunarea de Jos University of Galati, 111 Domneasca Street, Galati 800201, Romania; n Department of Mathematics and Applied Mathematics, Sefako Makgatho Health Sciences University, Medunsa 0204, South Africa

**Keywords:** Solitons, White noise, Integrability

## Abstract

This paper investigates new optical soliton solutions to the complex Ginzburg–Landau equation in the presence of white noise, a fundamental model in nonlinear optics that describes soliton dynamics. The study focuses on nine distinct forms of self-phase modulation structures, each exhibiting unique nonlinear characteristics and dispersion properties. To derive the soliton solutions, the generalized $G^{\prime }/G$-expansion approach is employed, which is known for its effectiveness in handling nonlinear differential equations and extracting exact solutions systematically. Through this analytical framework, a variety of soliton profiles are retrieved, demonstrating the influence of nonlinear dispersion and gain-loss terms on soliton propagation. A key observation from the analysis is that the presence of white noise primarily affects the phase component of the solitons, while their amplitude remains intact. This result suggests that the robustness of the soliton amplitude against stochastic perturbations could have significant implications for practical optical communication systems and laser pulse propagation, where maintaining stable intensity profiles is crucial. The obtained results provide valuable insights into the interplay between noise and nonlinear wave dynamics, offering potential applications in fiber-optic communication, mode-locked lasers, and other areas of photonics where controlled soliton evolution is essential.

• The paper investigates optical soliton solutions of the complex Ginzburg–Landau equation with white noise, focusing on nine forms of self-phase modulation with varied nonlinear and dispersive properties.
• Using the generalized $G^{\prime }/G$ -expansion method, the study derives exact soliton profiles, revealing how nonlinear dispersion and gain-loss mechanisms shape soliton dynamics.
• A key finding shows that white noise affects soliton phase without disturbing amplitude, highlighting amplitude robustness with implications for optical communications and laser systems.

## Graphic abstract

**Figure:**
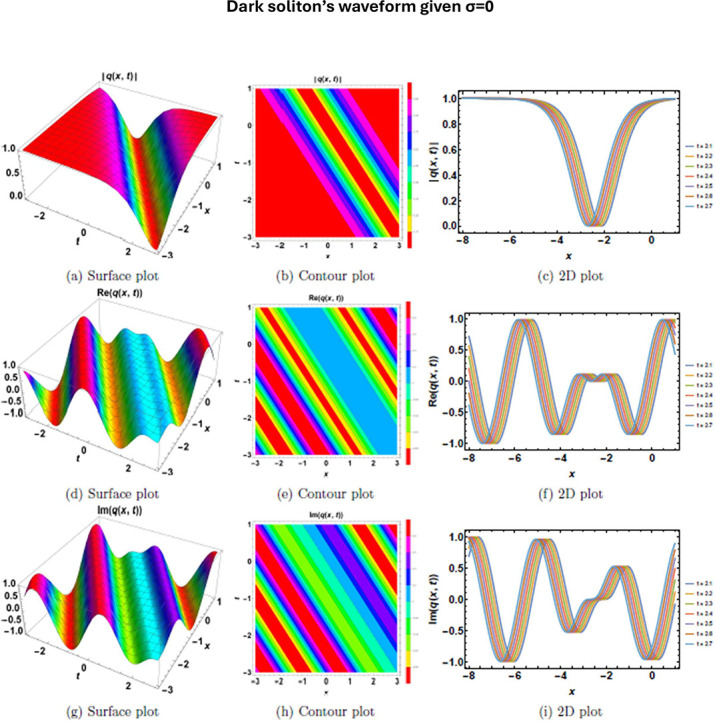

**Specifications table**

**Table:** 

**Subject area**	Mathematics and Statistics
**More specific subject area**	Mathematical Physics
**Name of your method**	The generalized $G^{\prime }/G$-expansion approach.
**Name and reference of original method**	E. M. E. Zayed. “New traveling wave solutions for higher dimensional nonlinear evolution equations using a generalized $G^{\prime }/G$–expansion method". *Journal of Physics A: Mathematical and Theoretical*. Volume 42, 195,202. (2009).
**Resource availability**	Mathematica & Maple

## Background

In this study, we introduce a perturbed complex Ginzburg–Landau equation (CGLE) incorporating multiplicative white noise in the Itô sense for the first time, expressed as:

(1) $$\begin{array}{c} iq_{t}+aq_{xx}+F\left(|q{|}^{2}\right)q+\sigma q\frac{dW\left(t\right)}{dt}=\frac{1}{|q{|}^{2}q^{*}}\left\{\alpha |q{|}^{2}{\left(|q{|}^{2}\right)}_{xx}-\beta {\left[{\left(|q{|}^{2}\right)}_{x}\right]}^{2}\right\}\\ +\gamma q+i\left[\lambda_{1}q_{x}+\mu_{1}{\left(|q{|}^{2}q\right)}_{x}+\theta_{1}{\left(|q{|}^{2}\right)}_{x}q+\upsilon_{1}|q{|}^{2}q_{x}\right], \end{array}$$

where q(x,t) represents the evolution of optical pulses. The term $iq_{t}$ represents the linear temporal evolution of the wave function, where $i=\sqrt{-1}$. The term $aq_{xx}$ describes chromatic dispersion [[1], [2], [3]]. The term $F(|q{|}^{2})q$ introduces nonlinear effects. The terms involving α and β account for modifications to the nonlinear dispersion [[4], [5], [6]]. The term γq represents detuning effects [[7], [8], [9]]. The term $\lambda_{1}q_{x}$ captures coupling effects. The term $\mu_{1}{\left(|q{|}^{2}q\right)}_{x}$ accounts for self-steepening [[10], [11], [12]]. The terms $\theta_{1}{\left(|q{|}^{2}\right)}_{x}q$ and $\upsilon_{1}|q{|}^{2}q_{x}$ describe higher-order dispersive and nonlinear interactions [[13], [14], [15]]. The term σ represents the strength of the noise, while W(t) is a Wiener process, with dW(t)/dt corresponding to Gaussian white noise [[16], [17], [18], [19]].

The core objective of this work is to analyze Eq. (1) and derive exact soliton solutions using the generalized $\left(G^{\prime }/G\right)$-expansion method [[20], [21], [22]], which is a robust analytical technique effective in constructing a wide class of localized wave structures. We explore nine distinct nonlinearities for $F(|q{|}^{2})$, enabling the identification of dark, singular, and mixed soliton solutions under varying physical conditions.

The novelty of this study lies in addressing, for the first time, a perturbed CGLE that incorporates white noise and multiple self-phase modulation (SPM) structures. In contrast to previous research centered on deterministic models [[1], [2], [3], [4], [5], [6], [7], [8], [9], [10], [11], [12], [13], [14], [15]], this work systematically investigates how stochastic perturbations influence soliton propagation. By considering nine distinct nonlinear forms, the study offers a broad perspective on the effects of nonlinearities and dispersion on soliton dynamics [[23], [24], [25]]. A key result shows that white noise alters the phase of solitons without affecting their amplitude, indicating that solitons can maintain stable intensity profiles in noisy environments. This property is particularly important for optical communication and pulse transmission [[26], [27], [28]]. The interaction between gain-loss terms and nonlinear dispersion is also analyzed, providing practical insights into the stability and evolution of solitons. The results have direct applications in fiber-optic systems [29], mode-locked lasers [30], and related photonic technologies [31]. Additionally, the use of the generalized $\left(G^{\prime }/G\right)$-expansion method demonstrates its capability to extract exact solutions in nonlinear systems with stochastic effects. Thus, this study advances the theoretical understanding of soliton behavior under noise and contributes to the development of reliable models for real-world optical systems [[32], [33], [34]].

We introduce the following wave transformation to analyze the optical soliton solutions of the CGLE in the presence of white noise:

(2) $$q\left(x,t\right)=\phi \left(\xi \right)e^{i\left[-kx+\omega t-\sigma^{2}t+\sigma W\left(t\right)+\theta_{0}\right]},$$

where q(x,t) represents the complex wave function describing the soliton profile. In this transformation, k is the soliton frequency, determining the oscillatory behavior of the wave in space, while ω corresponds to the wave number, governing the temporal oscillations. The term $\theta_{0}$ represents a phase constant, allowing for an initial phase shift in the soliton solution. The function ϕ(ξ) is a real-valued function that defines the soliton’s pulse shape and depends on the traveling wave variable ξ, given by

(3) ξ=x−vt,

where v denotes the soliton velocity. This transformation converts the original partial differential equation into an ordinary differential equation in terms of ξ, simplifying the analysis of soliton structures. Additionally, the presence of white noise is incorporated through the stochastic term σW(t), where W(t) represents a standard Wiener process and σ characterizes the intensity of the noise. The term $-\sigma^{2}t$ accounts for the Ito correction arising from the stochastic nature of the phase evolution. Notably, the stochastic influence remains confined to the phase component of the soliton, ensuring that the amplitude function ϕ(ξ) remains deterministic. This observation is crucial for understanding the robustness of soliton propagation under random perturbations and has potential implications in optical communication and nonlinear wave dynamics. Inserting (2) along with (3) into Eq. (1) we get:

(4) $$\begin{array}{c} -iv\phi \phi^{\prime }-\left(\omega -\sigma^{2}\right)\phi^{2}+a\left[\phi \phi^{\prime \prime }-2ik\phi \phi^{\prime }-k^{2}\phi^{2}\right]\\ +F\left(\phi^{2}\right)\phi^{2}-2\alpha \left(\phi \phi^{\prime \prime }+\phi^{{}^{\prime }2}\right)+4\beta \phi^{{}^{\prime }2}-\gamma \phi^{2}-i[\lambda_{1}\left(\phi \phi^{\prime }-ik\phi^{2}\right)+\mu_{1}\left(3\phi^{\prime }-ik\phi \right)\phi^{3}\\ +2\;\theta_{1}\phi^{3}\phi^{\prime }+\upsilon_{1}\left(\phi^{\prime }-ik\phi \right)\phi^{3}]=0. \end{array}$$

From Eq. (4), we have the imaginary part as:

(5) $$\left[-v-2ak-\lambda_{1}\right]\phi \phi^{\prime }-\left[3\mu_{1}+2\theta_{1}+\upsilon_{1}\right]\phi^{3}\phi^{\prime }=0.$$

On applying the linearly independence on Eq. (5), yields the soliton velocity:

(6) $$v=-2ak-\lambda_{1},$$

along with

(7) $$3\mu_{1}+2\;\theta_{1}+\upsilon_{1}=0.$$

Eq. (7) provides the constraint that governs the relationship among the perturbation terms appearing in Eq. (1), specifically accounting for self-steepening, higher-order dispersive, and nonlinear interaction effects. From Eq. (4), we have the real part as:

(8) $$\begin{array}{c} \left(a-2\alpha \right)\phi \phi^{\prime \prime }-\left[ak^{2}+\omega -\sigma^{2}+\gamma +\lambda_{1}k\right]\phi^{2}+F\left(\phi^{2}\right)\phi^{2}\\ +2\left(2\beta -\alpha \right)\phi^{{}^{\prime }2}-k\left(\mu_{1}+\upsilon_{1}\right)\phi^{4}=0. \end{array}$$

## Method details

Consider a model equation:

(9) $$F\left(u,u_{x},u_{xx},u_{t},u_{tt},\ldots \right)=0.$$

where F is a polynomial of u(x,t) and its partial derivatives.

Step 1**:** The restriction:

(10) u(x,t)=ϕ(ξ),ξ=x−ct,

changes Eq. (9) to

(11) $$P\left(\phi ,\phi^{\prime },\phi^{\prime \prime },\ldots \right)=0,$$

where ${}^{\prime }=\frac{d}{d\xi }$ and c is a constant.

Step 2**:**Eq. (11) holds:

(12) $$\phi \left(\xi \right)=\sum_{j=0}^{N}\;A_{j}{\left(\frac{G^{\prime }}{G}\right)}^{j}\;A_{N}\neq 0,$$

where

(13) $${\left(G^{\prime }\left(\xi \right)\right)}^{2}=e_{2}G^{4}\left(\xi \right)+e_{1}G^{2}\left(\xi \right)+e_{0},\;e_{2}\neq 0.$$

Here $A_{j}\;\left(j=0,1,\ldots ,N\right),e_{0},e_{1}$ and $e_{2}$ are constants. Also Eq. (13) satisfies [12,13,35]:

**Table:** 

case	$e_{0}$	$e_{1}$	$e_{2}$	G(ξ)	$G^{\prime }\left(\xi \right)$
1 2 3 4 5 6 7 8 9 10 11 12 13 14 15 16 17	1 1 $1-m^{2}$ $m^{2}-1$ $m^{2}$ $m^{2}$ $-m^{2}$ −1 $1-m^{2}$ 1 1 $m^{2}\left(m^{2}-1\right)$ 0.25 $0.25(1-m^{2})$ $0.25\;m^{2}$ 0 0	$-(1+m^{2})$ $-(1+m^{2})$ $2m^{2}-1$ $2-m^{2}$ $-(m^{2}+1)$ $-(m^{2}+1)$ $2m^{2}-1$ $2-m^{2}$ $2-m^{2}$ $2-m^{2}$ $2m^{2}-1$ $2m^{2}-1$ $0.5(1-2m^{2})$ $0.5(1+m^{2})$ $0.5(m^{2}-2)$ 1 1	$m^{2}$ $m^{2}$ $-m^{2}$ −1 1 1 $1-m^{2}$ $m^{2}-1$ 1 $1-m^{2}$ $m^{2}\left(m^{2}-1\right)$ 1 0.25 $0.25(1-m^{2})$ 0.25 −1 1	sn(ξ) cd(ξ) cn(ξ) dn(ξ) ns(ξ) dc(ξ) nc(ξ) nd(ξ) cs(ξ) sc(ξ) sd(ξ) ds(ξ) ns(ξ)±cs(ξ) nc(ξ)±sc(ξ) ns(ξ)±ds(ξ) sech(ξ) csch(ξ)	cn(ξ)dn(ξ) $-\left(1-m^{2}\right)sd\left(\xi \right)nd\left(\xi \right)$ −sn(ξ)dn(ξ) $-m^{2}sn\left(\xi \right)cn\left(\xi \right)$ −ds(ξ)cs(ξ) $\left(1-m^{2}\right)nc\left(\xi \right)sc\left(\xi \right)$ sc(ξ)dc(ξ) $m^{2}sd\left(\xi \right)cd\left(\xi \right)$ −ns(ξ)ds(ξ) nc(ξ)dc(ξ) nd(ξ)cd(ξ) −cs(ξ)ns(ξ) −ds(ξ)cs(ξ)∓ns(ξ)ds(ξ) sc(ξ)dc(ξ)±nc(ξ)dc(ξ) −ds(ξ)cs(ξ)∓cs(ξ)ns(ξ) −sech(ξ)tanh(ξ) −csch(ξ)coth(ξ)

where 0<m<1.

Step 3**:** Substituting Eqs. (12) and (13) into Eq. (11) and equating the coefficients of each power of $G^{l_{1}}\left(\xi \right)G^{l_{2}}\left(\xi \right)$$\left(l_{1}=0,\pm 1,\pm 2,\ldots ,\pm 4,l_{2}=0,1\right)$ to zero, we derive a solvable system of algebraic equations.

In the case of Kerr-law nonlinearity, we arrive at:

(14) $$F\left(|q{|}^{2}\right)=c|q{|}^{2},$$

where c is a constant. Also, Eq. (1) becomes:

(15) $$\begin{array}{c} iq_{t}+aq_{xx}+c|q{|}^{2}q+\sigma q\frac{dW\left(t\right)}{dt}=\frac{1}{|q{|}^{2}q^{*}}\left\{\alpha |q{|}^{2}{\left(|q{|}^{2}\right)}_{xx}-\beta {\left[{\left(|q{|}^{2}\right)}_{x}\right]}^{2}\right\}\\ +\gamma q+i\left[\lambda_{1}q_{x}+\mu_{1}{\left(|q{|}^{2}q\right)}_{x}+\theta_{1}{\left(|q{|}^{2}\right)}_{x}q+\upsilon_{1}|q{|}^{2}q_{x}\right], \end{array}$$

while Eq. (8) simplifies to

(16) $$\begin{array}{c} \left(a-2\alpha \right)\phi \phi^{\prime \prime }-\left[ak^{2}+\omega -\sigma^{2}+\gamma +\lambda_{1}k\right]\phi^{2}\\ +2\left(2\beta -\alpha \right)\phi^{{}^{\prime }2}-\left[k\left(\mu_{1}+\upsilon_{1}\right)-c\right]\phi^{4}=0. \end{array}$$

Balancing $\phi^{4}$ with $\phi \phi^{\prime \prime }$ in Eq. (16) leads to N=1. Then Eq. (12) comes out as

(17) $$\phi \left(\xi \right)=A_{0}+A_{1}\left(\frac{G^{\prime }}{G}\right),\;A_{1}\neq 0.$$

Substituting (17) along with (13) into Eq. (16) causes to

$$A_{0}=0,\;A_{1}=\sqrt{\frac{-\sigma^{2}+\omega +a\;k^{2}+k\lambda_{1}+\gamma }{e_{1}\left(c-k\mu_{1}-k\upsilon_{1}\right)}},$$

along with the constraint conditions:

(18) $$\begin{array}{c} \alpha =\frac{a\;k^{2}+\lambda_{1}k+2ae_{1}-\sigma^{2}+\gamma +\omega }{4e_{1}},\\ \beta =\frac{a\;k^{2}+\lambda_{1}k+2ae_{1}-\sigma^{2}+\gamma +\omega }{8e_{1}}, \end{array}$$

and

$e_{1}\left[-\sigma^{2}+\omega +a\;k^{2}+k\lambda_{1}+\gamma \right]\left(c-k\mu_{1}-k\upsilon_{1}\right)>0.$

Substituting (18) along with (17) into (2), we get

(19) $$q\left(x,t\right)=\sqrt{\frac{-\sigma^{2}+\omega +a\;k^{2}+k\lambda_{1}+\gamma }{e_{1}\left(c-k\mu_{1}-k\upsilon_{1}\right)}}\left(\frac{G^{\prime }}{G}\right)e^{i\left[-kx+\omega t-\sigma^{2}t+\sigma W\left(t\right)+\theta_{0}\right]}.$$

*Case 1***:** Setting G(ξ)=sn(ξ) or G(ξ)=cd(ξ), $e_{0}=1,e_{2}=m^{2},e_{1}=-\left(m^{2}+1\right)$ paves way to

(20) $$q\left(x,t\right)=\sqrt{-\frac{-\sigma^{2}+\omega +a\;k^{2}+k\lambda_{1}+\gamma }{\left(m^{2}+1\right)\left(c-k\mu_{1}-k\upsilon_{1}\right)}}\;\left[\frac{cn\left(\xi \right)dn\left(\xi \right)}{sn\left(\xi \right)}\right]e^{i\left[-kx+\omega t-\sigma^{2}t+\sigma W\left(t\right)+\theta_{0}\right]},$$

or

(21) $$q\left(x,t\right)=-\sqrt{-\frac{-\sigma^{2}+\omega +a\;k^{2}+k\lambda_{1}+\gamma }{\left(m^{2}+1\right)\left(c-k\mu_{1}-k\upsilon_{1}\right)}}\;\left[\left(1-m^{2}\right)\left(\frac{sd\left(\xi \right)nd\left(\xi \right)}{cd\left(\xi \right)}\right)\right]e^{i\left[-kx+\omega t-\sigma^{2}t+\sigma W\left(t\right)+\theta_{0}\right]},$$

provided $\left[-\sigma^{2}+\omega +a\;k^{2}+k\lambda_{1}+\gamma \right]\left(c-k\mu_{1}-k\upsilon_{1}\right)<0$.

A straddled soliton is presented by $m\to 1^{-}$ in Eq. (20):

(22) $$q\left(x,t\right)=\sqrt{\frac{-\sigma^{2}+\omega +a\;k^{2}+\lambda_{1}k+\gamma }{2\left(\mu_{1}+\upsilon_{1}\right)k-2c}}\;\left[\text{coth}\left(\xi \right)-\text{tanh}\left(\xi \right)\right]e^{i\left[-kx+\omega t-\sigma^{2}t+\sigma W\left(t\right)+\theta_{0}\right]},$$

provided $\left[-\sigma^{2}+\omega +a\;k^{2}+\lambda_{1}k+\gamma \right]\left[(\mu_{1}+\upsilon_{1})k-c\right]>0$.

*Case 2***:** Assuming G(ξ)=cn(ξ), $e_{0}=1-m^{2},e_{2}=-m^{2},e_{1}=2m^{2}-1$ causes to

(23) $$q\left(x,t\right)=-\sqrt{\frac{-\sigma^{2}+\omega +a\;k^{2}+k\lambda_{1}+\gamma }{\left(2m^{2}-1\right)\left(c-k\mu_{1}-k\upsilon_{1}\right)}}\left[\frac{sn\left(\xi \right)dn\left(\xi \right)}{cn\left(\xi \right)}\right]e^{i\left[-kx+\omega t-\sigma^{2}t+\sigma W\left(t\right)+\theta_{0}\right]},$$

provided $\left[-\sigma^{2}+\omega +a\;k^{2}+k\lambda_{1}+\gamma \right]\left(c-k\mu_{1}-k\upsilon_{1}\right)\left(2m^{2}-1\right)>0$.

A dark soliton is formulated by $m\to 1^{-}$:

(24) $$q\left(x,t\right)=-\sqrt{\frac{-\sigma^{2}+\omega +a\;k^{2}+k\lambda_{1}+\gamma }{c-k\mu_{1}-k\upsilon_{1}}}\;\text{tanh}\left(\xi \right)e^{i\left[-kx+\omega t-\sigma^{2}t+\sigma W\left(t\right)+\theta_{0}\right]},$$

provided $\left[-\sigma^{2}+\omega +a\;k^{2}+k\lambda_{1}+\gamma \right]\left(c-k\mu_{1}-k\upsilon_{1}\right)>0$.

*Case 3***:** Taking G(ξ)=dn(ξ), $e_{0}=m^{2}-1,e_{2}=-1,e_{1}=2-m^{2}$ leaves us with

(25) $$q\left(x,t\right)=-\sqrt{\frac{-\sigma^{2}+\omega +a\;k^{2}+k\lambda_{1}+\gamma }{\left(2-m^{2}\right)\left(c-k\mu_{1}-k\upsilon_{1}\right)}}\left[m^{2}\left(\frac{sn\left(\xi \right)cn\left(\xi \right)}{dn\left(\xi \right)}\right)\right]e^{i\left[-kx+\omega t-\sigma^{2}t+\sigma W\left(t\right)+\theta_{0}\right]},$$

provided

$\left[-\sigma^{2}+\omega +a\;k^{2}+k\lambda_{1}+\gamma \right]\left(c-k\mu_{1}-k\upsilon_{1}\right)>0.$

Here, the dark soliton (24) is extracted by $m\to 1^{-}$.

*Case 4***:** Choosing G(ξ)=ns(ξ) or G(ξ)=dc(ξ), $e_{0}=m^{2},e_{2}=1,e_{1}=-\left(m^{2}+1\right)$ allows us

(26) $$q\left(x,t\right)=-\sqrt{-\frac{-\sigma^{2}+\omega +a\;k^{2}+k\lambda_{1}+\gamma }{\left(m^{2}+1\right)\left(c-k\mu_{1}-k\upsilon_{1}\right)}}\left[\frac{ds\left(\xi \right)cs\left(\xi \right)}{ns\left(\xi \right)}\right]e^{i\left[-kx+\omega t-\sigma^{2}t+\sigma W\left(t\right)+\theta_{0}\right]},$$

or

(27) $$q\left(x,t\right)=\sqrt{-\frac{-\sigma^{2}+\omega +a\;k^{2}+k\lambda_{1}+\gamma }{\left(m^{2}+1\right)\left(c-k\mu_{1}-k\upsilon_{1}\right)}}\left[\left(1-m^{2}\right)\left(\frac{nc\left(\xi \right)sc\left(\xi \right)}{dc\left(\xi \right)}\right)\right]e^{i\left[-kx+\omega t-\sigma^{2}t+\sigma W\left(t\right)+\theta_{0}\right]},$$

provided $\left[-\sigma^{2}+\omega +a\;k^{2}+k\lambda_{1}+\gamma \right]\left(c-k\mu_{1}-k\upsilon_{1}\right)<0$.

A straddled soliton is constructed by $m\to 1^{-}$ in (26):

(28) $$q\left(x,t\right)=-\sqrt{\frac{-\sigma^{2}+\omega +a\;k^{2}+\lambda_{1}k+\gamma }{2\left(\mu_{1}+\upsilon_{1}\right)k-2c}}\;\left[\text{coth}\left(\xi \right)-\text{tanh}\left(\xi \right)\right]e^{i\left[-kx+\omega t-\sigma^{2}t+\sigma W\left(t\right)+\theta_{0}\right]},$$

provided $\left[-\sigma^{2}+\omega +a\;k^{2}+\lambda_{1}k+\gamma \right]\left[(\mu_{1}+\upsilon_{1})k-c\right]>0$.

*Case 5***:** Taking G(ξ)=nd(ξ), $e_{0}=-1,e_{2}=m^{2}-1,e_{1}=2-m^{2}$ paves way to

(29) $$q\left(x,t\right)=\sqrt{\frac{-\sigma^{2}+\omega +a\;k^{2}+k\lambda_{1}+\gamma }{\left(2-m^{2}\right)\left(c-k\mu_{1}-k\upsilon_{1}\right)}}\left[m^{2}\left(\frac{sd\left(\xi \right)cd\left(\xi \right)}{nd\left(\xi \right)}\right)\right]e^{i\left[-kx+\omega t-\sigma^{2}t+\sigma W\left(t\right)+\theta_{0}\right]}.$$

A dark soliton is recovered by $m\to 1^{-}$:

(30) $$q\left(x,t\right)=\sqrt{\frac{-\sigma^{2}+\omega +a\;k^{2}+\lambda_{1}k+\gamma }{c-k\mu_{1}-k\upsilon_{1}}}\;\text{tanh}\left(\xi \right)e^{i\left[-kx+\omega t-\sigma^{2}t+\sigma W\left(t\right)+\theta_{0}\right]},$$

provided $\left[-\sigma^{2}+\omega +a\;k^{2}+k\lambda_{1}+\gamma \right]\left(c-k\mu_{1}-k\upsilon_{1}\right)>0$.

*Case 6***:** Choosing G(ξ)=sc(ξ), $e_{0}=1,e_{2}=1-m^{2}e_{2}=1-m^{2},e_{1}=2-m^{2}$ leaves us with

(31) $$q\left(x,t\right)=\sqrt{\frac{-\sigma^{2}+\omega +a\;k^{2}+k\lambda_{1}+\gamma }{\left(2-m^{2}\right)\left(c-k\mu_{1}-k\upsilon_{1}\right)}}\left[\frac{nc\left(\xi \right)dc\left(\xi \right)}{sc\left(\xi \right)}\right]e^{i\left[-kx+\omega t-\sigma^{2}t+\sigma W\left(t\right)+\theta_{0}\right]}.$$

A singular soliton is defined by $m\to 1^{-}$:

(32) $$q\left(x,t\right)=\sqrt{\frac{-\sigma^{2}+\omega +a\;k^{2}+\lambda_{1}k+\gamma }{c-k\mu_{1}-k\upsilon_{1}}}\;\text{coth}\left(\xi \right)e^{i\left[-kx+\omega t-\sigma^{2}t+\sigma W\left(t\right)+\theta_{0}\right]},$$

provided $\left[-\sigma^{2}+\omega +a\;k^{2}+k\lambda_{1}+\gamma \right]\left(c-k\mu_{1}-k\upsilon_{1}\right)>0$.

Case 7**:** Taking G(ξ)=cs(ξ), $e_{0}=1-m^{2},e_{2}=1,e_{1}=2-m^{2},$ leaves us with

(33) $$q\left(x,t\right)=-\sqrt{\frac{-\sigma^{2}+\omega +a\;k^{2}+k\lambda_{1}+\gamma }{\left(2-m^{2}\right)\left(c-k\mu_{1}-k\upsilon_{1}\right)}}\left[\frac{ns\left(\xi \right)ds\left(\xi \right)}{cs\left(\xi \right)}\right]e^{i\left[-kx+\omega t-\sigma^{2}t+\sigma W\left(t\right)+\theta_{0}\right]}.$$

A singular soliton is recovered by $m\to 1^{-}$:

(34) $$q\left(x,t\right)=-\sqrt{\frac{-\sigma^{2}+\omega +a\;k^{2}+k\lambda_{1}+\gamma }{c-k\mu_{1}-k\upsilon_{1}}}\;\text{coth}\left(\xi \right)e^{i\left[-kx+\omega t-\sigma^{2}t+\sigma W\left(t\right)+\theta_{0}\right]},$$

provided $\left[-\sigma^{2}+\omega +a\;k^{2}+k\lambda_{1}+\gamma \right]\left(c-k\mu_{1}-k\upsilon_{1}\right)>0$.

*Case 8***:** Assuming G(ξ)=ns(ξ)±cs(ξ), $e_{0}=\frac{1}{4},e_{2}=\frac{1}{4},e_{1}=\frac{1}{2}\left(1-2m^{2}\right)$ leads to

(35) $$q\left(x,t\right)=\sqrt{-\frac{2\left[-\sigma^{2}+\omega +a\;k^{2}+k\lambda_{1}+\gamma \right]}{\left(2m^{2}-1\right)\left(c-k\mu_{1}-k\upsilon_{1}\right)}}ds\left(\xi \right)\;e^{i\left[-kx+\omega t-\sigma^{2}t+\sigma W\left(t\right)+\theta_{0}\right]},$$

provided $\left[-\sigma^{2}+\omega +a\;k^{2}+k\lambda_{1}+\gamma \right]\left(c-k\mu_{1}-k\upsilon_{1}\right)\left(2m^{2}-1\right)<0$.

A singular soliton is formulated by $m\to 1^{-}$:

(36) $$q\left(x,t\right)=\sqrt{-\frac{2\left[-\sigma^{2}+\omega +a\;k^{2}+\lambda_{1}k+\gamma \right]}{c-k\mu_{1}-k\upsilon_{1}}}\;\text{csch}\left(\xi \right)e^{i\left[-kx+\omega t-\sigma^{2}t+\sigma W\left(t\right)+\theta_{0}\right]},$$

provided $\left[-\sigma^{2}+\omega +a\;k^{2}+k\lambda_{1}+\gamma \right]\left(c-k\mu_{1}-k\upsilon_{1}\right)<0$.

*Case 9***:** Choosing G(ξ)=nc(ξ)±sc(ξ), $e_{0}=\frac{1}{4}\left(1-m^{2}\right),e_{2}=\frac{1}{4}\left(1-m^{2}\right),e_{1}=\frac{1}{2}\left(1+m^{2}\right)$ provides us

(37) $$q\left(x,t\right)=\sqrt{\frac{2\left[-\sigma^{2}+\omega +a\;k^{2}+k\lambda_{1}+\gamma \right]}{\left[c-\left(\upsilon_{1}+\mu_{1}\right)k\right]\left(m^{2}+1\right)}}dc\left(\xi \right)\;e^{i\left[-kx+\omega t-\sigma^{2}t+\sigma W\left(t\right)+\theta_{0}\right]},$$

provided $\left[-\sigma^{2}+\omega +a\;k^{2}+k\lambda_{1}+\gamma \right]\left[c-(\upsilon_{1}+\mu_{1})k\right]>0$.

*Case 10***:** Taking G(ξ)=ns(ξ)±ds(ξ), $e_{0}=\frac{m^{2}}{4},e_{2}=\frac{1}{4},e_{1}=\frac{1}{2}\left(m^{2}-2\right)$ causes to

(38) $$q\left(x,t\right)=\sqrt{\frac{2\left[-\sigma^{2}+\omega +a\;k^{2}+k\lambda_{1}+\gamma \right]}{\left[c-\left(\upsilon_{1}+\mu_{1}\right)k\right]\left(m^{2}-2\right)}}cs\left(\xi \right)\;e^{i\left[-kx+\omega t-\sigma^{2}t+\sigma W\left(t\right)+\theta_{0}\right]},$$

provided $\left[-\sigma^{2}+\omega +a\;k^{2}+k\lambda_{1}+\gamma \right]\left[c-(\upsilon_{1}+\mu_{1})k\right]\left(m^{2}-2\right)>0$.

Here, the singular soliton (36) is recovered by $m\to 1^{-}$.

In the case of Power-law nonlinearity, we arrive at:

(39) $$F\left({\left|q\right|}^{2}\right)=c\;{\left|q\right|}^{2n},$$

where c is a constant. Also, Eq. (1) turns into

(40) $$\begin{array}{c} iq_{t}+aq_{xx}+c\;|q{|}^{2n}q+\sigma q\frac{dW\left(t\right)}{dt}=\frac{1}{|q{|}^{2}q^{*}}\left\{\alpha |q{|}^{2}{\left(|q{|}^{2}\right)}_{xx}-\beta {\left[{\left(|q{|}^{2}\right)}_{x}\right]}^{2}\right\}\\ +\gamma q+i\left[\lambda_{1}q_{x}+\mu_{1}{\left(|q{|}^{2}q\right)}_{x}+\theta_{1}{\left(|q{|}^{2}\right)}_{x}q+\upsilon_{1}|q{|}^{2}q_{x}\right], \end{array}$$

while Eq. (8) changes to

(41) $$\begin{array}{c} \left(a-2\alpha \right)\phi \phi^{\prime \prime }-\left[ak^{2}+\omega -\sigma^{2}+\gamma +\lambda_{1}k\right]\phi^{2}+c\phi^{2n+2}\\ +2\left(2\beta -\alpha \right)\phi^{{}^{\prime }2}-k\left(\mu_{1}+\upsilon_{1}\right)\phi^{4}=0. \end{array}$$

Balancing $\phi^{2n+2}$ with $\phi \phi^{\prime \prime }$ in Eq. (41) provides us $N=\frac{1}{n}$. Consider the restriction:

(42) $$\phi \left(\xi \right)={\left[\psi \left(\xi \right)\right]}^{\frac{1}{n}},$$

Thus, substituting (42) into Eq. (41) leaves us with

(43) $$\begin{array}{c} n\left(a-2\alpha \right)\psi \psi^{\prime \prime }+\left[\left(-a+2\alpha \right)n+a-4\alpha +4\beta \right]\psi^{{}^{\prime }2}+c\;n^{2}\psi^{4}\\ -\left[a\;k^{2}+-\sigma^{2}+\omega +\lambda_{1}k+\gamma \right]n^{2}\psi^{2}-k\;n^{2}\left(\mu_{1}+\upsilon_{1}\right)\psi^{\frac{2n+2}{n}}=0. \end{array}$$

For Eq. (43) to be integrated, we select $\mu_{1}+\upsilon_{1}=0$. Thus, Eq. (43) simplifies to

(44) $$\begin{array}{c} n\left(a-2\alpha \right)\psi \psi^{\prime \prime }+\left[\left(-a+2\alpha \right)n+a-4\alpha +4\beta \right]\psi^{{}^{\prime }2}+c\;n^{2}\psi^{4}\\ -\left[a\;k^{2}+-\sigma^{2}+\omega +\lambda_{1}k+\gamma \right]n^{2}\psi^{2}=0. \end{array}$$

Balancing $\psi \psi^{\prime \prime }$ and $\psi^{4}$ in Eq. (44) leads to N=1. Then Eq. (12) comes out as

(45) $$\psi \left(\xi \right)=A_{0}+A_{1}\left(\frac{G^{\prime }}{G}\right),\;A_{1}\neq 0.$$

Substituting (45) along with (13) into Eq. (44) paves way to

$A_{0}=0,\;A_{1}=A_{1},$along with the constraint conditions

(46) $$\begin{array}{c} a=\frac{-\lambda_{1}k+c\;A_{1}^{2}e_{1}+\sigma^{2}-\gamma -\omega }{k^{2}},\\ \alpha =\frac{\left(A_{1}^{2}cn\right)k^{2}-2\lambda_{1}k+2c\;A_{1}^{2}e_{1}+2\sigma^{2}-2\gamma -2\omega }{4k^{2}},\\ \beta =\frac{\left[cn\left(2-n\right)A_{1}^{2}\right]k^{2}-2\lambda_{1}k+2c\;A_{1}^{2}e_{1}+2\sigma^{2}-2\gamma -2\omega }{8k^{2}}, \end{array}$$

where $A_{1}$ is an arbitrary constant.

Substituting (46) along with (42) and (45) into (2), we get

(47) $$q\left(x,t\right)={\left[A_{1}\left(\frac{G^{\prime }}{G}\right)\right]}^{\frac{1}{n}}e^{i\left[-kx+\omega t-\sigma^{2}t+\sigma W\left(t\right)+\theta_{0}\right]},$$

provided $A_{1}\left(\frac{G^{\prime }}{G}\right)>0$.

*Case 1***:** Taking G(ξ)=sn(ξ) or G(ξ)=cd(ξ), $e_{0}=1,e_{2}=m^{2},e_{1}=-\left(m^{2}+1\right)$ and causes to

(48) $$q\left(x,t\right)={\left[A_{1}\left(\frac{cn\left(\xi \right)dn\left(\xi \right)}{sn\left(\xi \right)}\right)\right]}^{\frac{1}{n}}e^{i\left[-kx+\omega t-\sigma^{2}t+\sigma W\left(t\right)+\theta_{0}\right]},$$

or

(49) $$q\left(x,t\right)={\left[\left(m^{2}-1\right)A_{1}\left(\frac{sd\left(\xi \right)nd\left(\xi \right)}{cd\left(\xi \right)}\right)\right]}^{\frac{1}{n}}e^{i\left[-kx+\omega t-\sigma^{2}t+\sigma W\left(t\right)+\theta_{0}\right]}.$$

A straddled soliton is recovered by $m\to 1^{-}$ in Eq. (48):

(50) $$q\left(x,t\right)={\left\{A_{1}\left[\text{coth}\left(\xi \right)-\text{tanh}\left(\xi \right)\right]\right\}}^{\frac{1}{n}}e^{i\left[-kx+\omega t-\sigma^{2}t+\sigma W\left(t\right)+\theta_{0}\right]}.$$

*Case 2***:** Choosing G(ξ)=cn(ξ), $e_{0}=1-m^{2},e_{2}=-m^{2},e_{1}=2m^{2}-1$ and gives rise to

(51) $$q\left(x,t\right)={\left[-A_{1}\left(\frac{sn\left(\xi \right)dn\left(\xi \right)}{cn\left(\xi \right)}\right)\right]}^{\frac{1}{n}}e^{i\left[-kx+\omega t-\sigma^{2}t+\sigma W\left(t\right)+\theta_{0}\right]}.$$

A dark soliton is structured by $m\to 1^{-}$:

(52) $$q\left(x,t\right)={\left[-A_{1}\text{tanh}\left(\xi \right)\right]}^{\frac{1}{n}}e^{i\left[-kx+\omega t-\sigma^{2}t+\sigma W\left(t\right)+\theta_{0}\right]}.$$

*Case 3***:** Taking G(ξ)=dn(ξ), $e_{0}=m^{2}-1,e_{2}=-1,e_{1}=2-m^{2}$ provides us

(53) $$q\left(x,t\right)={\left[-m^{2}A_{1}\left(\frac{sn\left(\xi \right)cn\left(\xi \right)}{dn\left(\xi \right)}\right)\right]}^{\frac{1}{n}}e^{i\left[-kx+\omega t-\sigma^{2}t+\sigma W\left(t\right)+\theta_{0}\right]}.$$

The dark soliton (52) is structured by $m\to 1^{-}$.

*Case 4***:** Choosing G(ξ)=ns(ξ) or G(ξ)=dc(ξ), $e_{0}=m^{2},e_{2}=1,e_{1}=-\left(m^{2}+1\right)$ allows us

(54) $$q\left(x,t\right)={\left[-A_{1}\left(\frac{ds\left(\xi \right)cs\left(\xi \right)}{ns\left(\xi \right)}\right)\right]}^{\frac{1}{n}}e^{i\left[-kx+\omega t-\sigma^{2}t+\sigma W\left(t\right)+\theta_{0}\right]},$$

or

(55) $$q\left(x,t\right)={\left[\left(1-m^{2}\right)A_{1}\left(\frac{nc\left(\xi \right)sc\left(\xi \right)}{dc\left(\xi \right)}\right)\right]}^{\frac{1}{n}}e^{i\left[-kx+\omega t-\sigma^{2}t+\sigma W\left(t\right)+\theta_{0}\right]}.$$

A straddled soliton is extracted by $m\to 1^{-}$ in Eq. (54):

(56) $$q\left(x,t\right)={\left\{-A_{1}\left[\text{coth}\left(\xi \right)-\text{tanh}\left(\xi \right)\right]\right\}}^{\frac{1}{n}}e^{i\left[-kx+\omega t-\sigma^{2}t+\sigma W\left(t\right)+\theta_{0}\right]}.$$

*Case 5***:** Taking G(ξ)=nd(ξ), $e_{0}=-1,e_{2}=m^{2}-1,e_{1}=2-m^{2}$ provides us

(57) $$q\left(x,t\right)={\left[m^{2}A_{1}\left(\frac{sd\left(\xi \right)cd\left(\xi \right)}{nd\left(\xi \right)}\right)\right]}^{\frac{1}{n}}e^{i\left[-kx+\omega t-\sigma^{2}t+\sigma W\left(t\right)+\theta_{0}\right]}.$$

A dark soliton is extracted by $m\to 1^{-}$, as shown below

(58) $$q\left(x,t\right)={\left[A_{1}\text{tanh}\left(\xi \right)\right]}^{\frac{1}{n}}e^{i\left[-kx+\omega t-\sigma^{2}t+\sigma W\left(t\right)+\theta_{0}\right]}.$$

*Case 6***:** Choosing G(ξ)=sc(ξ), $e_{0}=1,e_{2}=1-m^{2},e_{1}=2-m^{2}$ leaves us with

(59) $$q\left(x,t\right)={\left[A_{1}\left(\frac{nc\left(\xi \right)dc\left(\xi \right)}{sc\left(\xi \right)}\right)\right]}^{\frac{1}{n}}e^{i\left[-kx+\omega t-\sigma^{2}t+\sigma W\left(t\right)+\theta_{0}\right]}.$$

A singular soliton is recovered by $m\to 1^{-}$:

(60) $$q\left(x,t\right)={\left[A_{1}\text{coth}\left(\xi \right)\right]}^{\frac{1}{n}}e^{i\left[-kx+\omega t-\sigma^{2}t+\sigma W\left(t\right)+\theta_{0}\right]}.$$

*Case 7***:** Taking G(ξ)=cs(ξ), $e_{0}=1-m^{2},e_{2}=1,e_{1}=2-m^{2}$ allows us

(61) $$q\left(x,t\right)={\left[-A_{1}\left(\frac{ns\left(\xi \right)ds\left(\xi \right)}{cs\left(\xi \right)}\right)\right]}^{\frac{1}{n}}e^{i\left[-kx+\omega t-\sigma^{2}t+\sigma W\left(t\right)+\theta_{0}\right]}.$$

A singular soliton is extracted by $m\to 1^{-}$:

(62) $$q\left(x,t\right)={\left[-A_{1}\text{coth}\left(\xi \right)\right]}^{\frac{1}{n}}e^{i\left[-kx+\omega t-\sigma^{2}t+\sigma W\left(t\right)+\theta_{0}\right]}.$$

*Case 8***:** Choosing G(ξ)=ns(ξ)±cs(ξ), $e_{0}=\frac{1}{4},e_{2}=\frac{1}{4},e_{1}=\frac{1}{2}\left(1-2m^{2}\right)$ yields:

(63) $$q\left(x,t\right)={\left[A_{1}ds\left(\xi \right)\right]}^{\frac{1}{n}}e^{i\left[-kx+\omega t-\sigma^{2}t+\sigma W\left(t\right)+\theta_{0}\right]}.$$

A singular soliton is extracted by $m\to 1^{-}$:

(64) $$q\left(x,t\right)={\left[A_{1}csch\left(\xi \right)\right]}^{\frac{1}{n}}e^{i\left[-kx+\omega t-\sigma^{2}t+\sigma W\left(t\right)+\theta_{0}\right]}.$$

*Case 9***:** Taking G(ξ)=nc(ξ)±sc(ξ), $e_{0}=\frac{1}{4}\left(1-m^{2}\right),e_{2}=\frac{1}{4}\left(1-m^{2}\right),e_{1}=\frac{1}{2}\left(1+m^{2}\right)$ causes to

(65) $$q\left(x,t\right)={\left[A_{1}dc\left(\xi \right)\right]}^{\frac{1}{n}}e^{i\left[-kx+\omega t-\sigma^{2}t+\sigma W\left(t\right)+\theta_{0}\right]}.$$

*Case 10***:** Choosing G(ξ)=ns(ξ)±ds(ξ), $e_{0}=\frac{m^{2}}{4},e_{2}=\frac{1}{4},e_{1}=\frac{1}{2}\left(m^{2}-2\right)$ gives rise to

(66) $$q\left(x,t\right)={\left[A_{1}cs\left(\xi \right)\right]}^{\frac{1}{n}}e^{i\left[-kx+\omega t-\sigma^{2}t+\sigma W\left(t\right)+\theta_{0}\right]}.$$

Here, the singular soliton (64) is recovered by $m\to 1^{-}$.

In the case of Parabolic-law nonlinearity, we arrive at:

(67) $$F\left(|q{|}^{2}\right)=k_{1}\left|q{|}^{2}+k_{2}\right|q{|}^{4},\;k_{2}\neq 0,$$

where $k_{1}$ and $k_{2}$ are constants. Also, Eq. (1) appears as

(68) $$\begin{array}{c} iq_{t}+aq_{xx}+\left(k_{1}\left|q{|}^{2}+k_{2}\right|q{|}^{4}\right)q+\sigma q\frac{dW\left(t\right)}{dt}=\frac{1}{|q{|}^{2}q^{*}}\left\{\alpha |q{|}^{2}{\left(|q{|}^{2}\right)}_{xx}-\beta {\left[{\left(|q{|}^{2}\right)}_{x}\right]}^{2}\right\}\\ +\gamma q+i\left[\lambda_{1}q_{x}+\mu_{1}{\left(|q{|}^{2}q\right)}_{x}+\theta_{1}{\left(|q{|}^{2}\right)}_{x}q+\upsilon_{1}|q{|}^{2}q_{x}\right], \end{array}$$

while Eq. (8) stands as

(69) $$\begin{array}{c} \left(a-2\alpha \right)\phi \phi^{\prime \prime }-\left[ak^{2}+\omega -\sigma^{2}+\gamma +\lambda_{1}k\right]\phi^{2}+k_{2}\phi^{6}\\ +2\left(2\beta -\alpha \right)\phi^{{}^{\prime }2}+\left[k_{1}-k\left(\mu_{1}+\upsilon_{1}\right)\right]\phi^{4}=0. \end{array}$$

Balancing $\phi^{6}$ with $\phi \phi^{\prime \prime }$ in Eq. (69) allows us $N=\frac{1}{2}$. Consider the relation:

(70) $$\phi \left(\xi \right)={\left[\psi \left(\xi \right)\right]}^{\frac{1}{2}},$$

Thus, plugging (70) into Eq. (69) yields:

(71) $$\begin{array}{c} 2\left(a-2\alpha \right)\psi \psi^{\prime \prime }+\left(-a+4\beta \right)\psi^{{}^{\prime }2}+4k_{2}\psi^{4}-4\left[\left(\mu_{1}+\upsilon_{1}\right)k-k_{1}\right]\psi^{3}\\ -4\left[a\;k^{2}+\omega -\sigma^{2}+\lambda_{1}k+\gamma \right]\psi^{2}=0. \end{array}$$

Balancing $\psi^{4}$ with $\psi \psi^{\prime \prime }$ in Eq. (71) leads to N=1. Lastly, Eq. (12) turns into

(72) $$\psi \left(\xi \right)=A_{0}+A_{1}\left(\frac{G^{\prime }}{G}\right),\;A_{1}\neq 0.$$

Inserting (72) along with (13) into Eq. (71) paves way to

$A_{0}=0,\;A_{1}=\sqrt{\frac{-\sigma^{2}+\omega +a\;k^{2}+k\lambda_{1}+\gamma }{e_{1}k_{2}}},$along with the constraint conditions

(73) $$\alpha =\frac{\omega -\sigma^{2}+a\;k^{2}+ae_{1}+k\lambda_{1}+\gamma }{2e_{1}},\;\beta =\frac{a}{4},\;\mu_{1}=-\frac{k\upsilon_{1}-k_{1}}{k},$$

provided $e_{1}k_{2}\left[-\sigma^{2}+\omega +a\;k^{2}+k\lambda_{1}+\gamma \right]>0$.

Substituting (73) along with (70) and (72) into (2), we get

(74) $$q\left(x,t\right)={\left\{\sqrt{\frac{-\sigma^{2}+\omega +a\;k^{2}+k\lambda_{1}+\gamma }{e_{1}k_{2}}}\left(\frac{G^{\prime }}{G}\right)\right\}}^{\frac{1}{2}}e^{i\left[-kx+\omega t-\sigma^{2}t+\sigma W\left(t\right)+\theta_{0}\right]},$$

provided $A_{1}\left(\frac{G^{\prime }}{G}\right)>0$.

*Case 1***:** Taking G(ξ)=sn(ξ) or G(ξ)=cd(ξ), $e_{0}=1,e_{2}=m^{2},e_{1}=-\left(m^{2}+1\right)$ yields

(75) $$q\left(x,t\right)={\left\{\sqrt{-\frac{-\sigma^{2}+\omega +a\;k^{2}+k\lambda_{1}+\gamma }{\left(m^{2}+1\right)k_{2}}}\left[\frac{cn\left(\xi \right)dn\left(\xi \right)}{sn\left(\xi \right)}\right]\right\}}^{\frac{1}{2}}e^{i\left[-kx+\omega t-\sigma^{2}t+\sigma W\left(t\right)+\theta_{0}\right]},$$

or

q(x,t)=

(76) $${\left\{-\sqrt{-\frac{-\sigma^{2}+\omega +a\;k^{2}+k\lambda_{1}+\gamma }{\left(m^{2}+1\right)k_{2}}}\left[\left(1-m^{2}\right)\left(\frac{sd\left(\xi \right)nd\left(\xi \right)}{cd\left(\xi \right)}\right)\right]\right\}}^{\frac{1}{2}}e^{i\left[-kx+\omega t-\sigma^{2}t+\sigma W\left(t\right)+\theta_{0}\right]}.$$

A straddled soliton is structured by $m\to 1^{-}$:

(77) $$q\left(x,t\right)={\left\{\sqrt{-\frac{-\sigma^{2}+\omega +a\;k^{2}+k\lambda_{1}+\gamma }{2k_{2}}}\;\left[\text{coth}\left(\xi \right)-\text{tanh}\left(\xi \right)\right]\right\}}^{\frac{1}{2}}e^{i\left[-kx+\omega t-\sigma^{2}t+\sigma W\left(t\right)+\theta_{0}\right]},$$

provided $k_{2}\left[-\sigma^{2}+\omega +a\;k^{2}+k\lambda_{1}+\gamma \right]<0$ and [coth(ξ)−tanh(ξ)]>0.

*Case 2***:** Choosing G(ξ)=nd(ξ), $e_{0}=-1,e_{2}=m^{2}-1,e_{1}=2-m^{2}$ gives

(78) $$q\left(x,t\right)={\left\{\sqrt{\frac{-\sigma^{2}+\omega +a\;k^{2}+k\lambda_{1}+\gamma }{\left(2-m^{2}\right)k_{2}}}\left[m^{2}\left(\frac{sd\left(\xi \right)cd\left(\xi \right)}{nd\left(\xi \right)}\right)\right]\right\}}^{\frac{1}{2}}e^{i\left[-kx+\omega t-\sigma^{2}t+\sigma W\left(t\right)+\theta_{0}\right]}.$$

A dark soliton is formulated by $m\to 1^{-}$:

(79) $$q\left(x,t\right)={\left\{\sqrt{\frac{-\sigma^{2}+\omega +a\;k^{2}+k\lambda_{1}+\gamma }{k_{2}}}\;\text{tanh}\left(\xi \right)\right\}}^{\frac{1}{2}}e^{i\left[-kx+\omega t-\sigma^{2}t+\sigma W\left(t\right)+\theta_{0}\right]},$$

provided $k_{2}\left[-\sigma^{2}+\omega +a\;k^{2}+k\lambda_{1}+\gamma \right]>0$ and tanh(ξ)>0.

*Case 3***:** Taking G(ξ)=sc(ξ), $e_{0}=1,e_{2}=1-m^{2},e_{1}=2-m^{2}$ leads to

(80) $$q\left(x,t\right)={\left\{\sqrt{\frac{-\sigma^{2}+\omega +a\;k^{2}+k\lambda_{1}+\gamma }{\left(2-m^{2}\right)k_{2}}}\left[\frac{nc\left(\xi \right)dc\left(\xi \right)}{sc\left(\xi \right)}\right]\right\}}^{\frac{1}{2}}e^{i\left[-kx+\omega t-\sigma^{2}t+\sigma W\left(t\right)+\theta_{0}\right]}.$$

A singular soliton is presented by $m\to 1^{-}$:

(81) $$q\left(x,t\right)={\left\{\sqrt{\frac{-\sigma^{2}+\omega +a\;k^{2}+k\lambda_{1}+\gamma }{k_{2}}}\;\text{coth}\left(\xi \right)\right\}}^{\frac{1}{2}}e^{i\left[-kx+\omega t-\sigma^{2}t+\sigma W\left(t\right)+\theta_{0}\right]},$$

provided $k_{2}\left[-\sigma^{2}+\omega +a\;k^{2}+k\lambda_{1}+\gamma \right]>0$ and coth(ξ)>0.

*Case 4***:** Choosing G(ξ)=ns(ξ)±cs(ξ), $e_{0}=\frac{1}{4},e_{2}=\frac{1}{4},e_{1}=\frac{1}{2}\left(1-2m^{2}\right)$ causes to

(82) $$q\left(x,t\right)={\left\{\sqrt{-\frac{2\left[-\sigma^{2}+\omega +a\;k^{2}+k\lambda_{1}+\gamma \right]}{\left(2m^{2}-1\right)k_{2}}}ds\left(\xi \right)\right\}}^{\frac{1}{2}}e^{i\left[-kx+\omega t-\sigma^{2}t+\sigma W\left(t\right)+\theta_{0}\right]},$$

provided $k_{2}\left[-\sigma^{2}+\omega +a\;k^{2}+k\lambda_{1}+\gamma \right]\left(2m^{2}-1\right)<0$.

A singular soliton is recovered by $m\to 1^{-}$:

(83) $$q\left(x,t\right)={\left\{\sqrt{-\frac{2\left[-\sigma^{2}+\omega +a\;k^{2}+k\lambda_{1}+\gamma \right]}{k_{2}}}csch\left(\xi \right)\right\}}^{\frac{1}{2}}e^{i\left[-kx+\omega t-\sigma^{2}t+\sigma W\left(t\right)+\theta_{0}\right]},$$

provided $k_{2}\left[-\sigma^{2}+\omega +a\;k^{2}+k\lambda_{1}+\gamma \right]<0$ and csch(ξ)>0.

*Case 5***:** Taking G(ξ)=nc(ξ)±sc(ξ), $e_{0}=\frac{1}{4}\left(1-m^{2}\right),e_{2}=\frac{1}{4}\left(1-m^{2}\right),e_{1}=\frac{1}{2}\left(1+m^{2}\right)$ gives rise to

(84) $$q\left(x,t\right)={\left\{\sqrt{\frac{2\left[-\sigma^{2}+\omega +a\;k^{2}+\lambda_{1}k+\gamma \right]}{\left(m^{2}+1\right)k_{2}}}dc\left(\xi \right)\right\}}^{\frac{1}{2}}e^{i\left[-kx+\omega t-\sigma^{2}t+\sigma W\left(t\right)+\theta_{0}\right]},$$

provided $k_{2}\left[-\sigma^{2}+\omega +a\;k^{2}+\lambda_{1}k+\gamma \right]>0$.

*Case 6***:** Choosing G(ξ)=ns(ξ)±ds(ξ), $e_{0}=\frac{m^{2}}{4},e_{2}=\frac{1}{4},e_{1}=\frac{1}{2}\left(m^{2}-2\right)$ paves way to

(85) $$q\left(x,t\right)={\left\{\sqrt{\frac{2\left[-\sigma^{2}+\omega +a\;k^{2}+\lambda_{1}k+\gamma \right]}{\left(m^{2}-2\right)k_{2}}}cs\left(\xi \right)\right\}}^{\frac{1}{2}}e^{i\left[-kx+\omega t-\sigma^{2}t+\sigma W\left(t\right)+\theta_{0}\right]},$$

provided

$k_{2}\left[-\sigma^{2}+\omega +a\;k^{2}+\lambda_{1}k+\gamma \right]\left(m^{2}-2\right)>0.$

Here, the singular soliton (83) is constructed by $m\to 1^{-}$.

In the case of Dual-law nonlinearity, we arrive at:

(86) $$F\left({\left|q\right|}^{2}\right)=k_{1}\left|q{|}^{2n}+k_{2}\right|q{|}^{2n+2},\;k_{2}\neq 0,$$

where $k_{1}$ and $k_{2}$ are constants. Also, Eq. (1) simplifies to

(87) $$\begin{array}{c} iq_{t}+aq_{xx}+\left(k_{1}\left|q{|}^{2n}+k_{2}\right|q{|}^{2n+2}\right)q+\sigma q\frac{dW\left(t\right)}{dt}=\frac{1}{|q{|}^{2}q^{*}}\left\{\alpha |q{|}^{2}{\left(|q{|}^{2}\right)}_{xx}-\beta {\left[{\left(|q{|}^{2}\right)}_{x}\right]}^{2}\right\}\\ +\gamma q+i\left[\lambda_{1}q_{x}+\mu_{1}{\left(|q{|}^{2}q\right)}_{x}+\theta_{1}{\left(|q{|}^{2}\right)}_{x}q+\upsilon_{1}|q{|}^{2}q_{x}\right], \end{array}$$

while Eq. (8) collapses to

(88) $$\begin{array}{c} \left(a-2\alpha \right)\phi \phi^{\prime \prime }-\left[ak^{2}+\omega -\sigma^{2}+\gamma +\lambda_{1}k\right]\phi^{2}+k_{1}\phi^{2n+2}+k_{2}\phi^{2n+4}\\ +2\left(2\beta -\alpha \right)\phi^{{}^{\prime }2}-k\left(\mu_{1}+\upsilon_{1}\right)\phi^{4}=0. \end{array}$$

Balancing $\phi^{2n+4}$ with $\phi \phi^{\prime \prime }$ in Eq. (88) allows us $N=\frac{1}{n+1}$. Consider the condition:

(89) $$\phi \left(\xi \right)={\left[\psi \left(\xi \right)\right]}^{\frac{1}{n+1}},$$

Substituting (89) into Eq. (88), we get

(90) $$\begin{array}{c} \left(n+1\right)\left(a-2\alpha \right)\psi \psi^{\prime \prime }-{\left(n+1\right)}^{2}\left[a\;k^{2}+\omega -\sigma^{2}+\lambda_{1}k+\gamma \right]\psi^{2}+{\left(n+1\right)}^{2}k_{1}\psi^{\frac{4n+2}{n+1}}\\ +{\left(n+1\right)}^{2}k_{2}\psi^{4}+\left[\left(2\alpha -a\right)n+4\beta -2\alpha \right]\psi^{{}^{\prime }2}-k{\left(n+1\right)}^{2}\left(\mu_{1}+\upsilon_{1}\right)\psi^{\frac{4+2n}{n+1}}=0. \end{array}$$

For Eq. (90) to be integrated, we select $k_{1}=0$ and $\mu_{1}+\upsilon_{1}=0$. Thus, Eq. (90) simplifies to

(91) $$\begin{array}{c} \left(n+1\right)\left(a-2\alpha \right)\psi \psi^{\prime \prime }-{\left(n+1\right)}^{2}\left[a\;k^{2}+\omega -\sigma^{2}+\lambda_{1}k+\gamma \right]\psi^{2}\\ +{\left(n+1\right)}^{2}k_{2}\psi^{4}+\left[\left(2\alpha -a\right)n+4\beta -2\alpha \right]\psi^{{}^{\prime }2}=0. \end{array}$$

Balancing $\psi \psi^{\prime \prime }$ and $\psi^{4}$ in Eq. (91) leads to N=1. Thus, Eq. (12) evolves as

(92) $$\psi \left(\xi \right)=A_{0}+A_{1}\left(\frac{G^{\prime }}{G}\right),\;A_{1}\neq 0.$$

Substituting (92) along with (13) into Eq. (91) leaves us with

$A_{0}=0,\;A_{1}=A_{1},$ along with the constraint conditions

(93) $$\begin{array}{c} k_{2}=\frac{a\;k^{2}+k\lambda_{1}-\sigma^{2}+\gamma +\omega }{A_{1}^{2}e_{1}},\;\;\;\;\alpha =\frac{\left(a\;k^{2}+k\lambda_{1}-\sigma^{2}+\gamma +\omega \right)\left(n+1\right)+2ae_{1}}{4e_{1}},\\ \beta =\frac{\left(a\;k^{2}+k\lambda_{1}-\sigma^{2}+\gamma +\omega \right)\left(1-n^{2}\right)+2ae_{1}}{8e_{1}}, \end{array}$$

where $A_{1}$ is an arbitrary constant.

Substituting (93) along with (89) and (92) into (2), we get

(94) $$q\left(x,t\right)={\left\{A_{1}\left(\frac{G^{\prime }}{G}\right)\right\}}^{\frac{1}{n+1}}e^{i\left[-kx+\omega t-\sigma^{2}t+\sigma W\left(t\right)+\theta_{0}\right]},$$

provided $A_{1}\left(\frac{G^{\prime }}{G}\right)>0$.

*Case 1***:** Taking G(ξ)=sn(ξ) or G(ξ)=cd(ξ), $e_{0}=1,e_{2}=m^{2},e_{1}=-\left(m^{2}+1\right)$ provides us

(95) $$q\left(x,t\right)={\left[A_{1}\left(\frac{cn\left(\xi \right)dn\left(\xi \right)}{sn\left(\xi \right)}\right)\right]}^{\frac{1}{n+1}}e^{i\left[-kx+\omega t-\sigma^{2}t+\sigma W\left(t\right)+\theta_{0}\right]},$$

or

(96) $$q\left(x,t\right)={\left[\left(m^{2}-1\right)A_{1}\left(\frac{sd\left(\xi \right)nd\left(\xi \right)}{cd\left(\xi \right)}\right)\right]}^{\frac{1}{n+1}}e^{i\left[-kx+\omega t-\sigma^{2}t+\sigma W\left(t\right)+\theta_{0}\right]}.$$

A straddled soliton is extracted by $m\to 1^{-}$ in Eq. (48):

(97) $$q\left(x,t\right)={\left\{A_{1}\left[\text{coth}\left(\xi \right)-\text{tanh}\left(\xi \right)\right]\right\}}^{\frac{1}{n+1}}e^{i\left[-kx+\omega t-\sigma^{2}t+\sigma W\left(t\right)+\theta_{0}\right]}.$$

*Case 2***:** Choosing G(ξ)=cn(ξ), $e_{0}=1-m^{2},e_{2}=-m^{2},e_{1}=2m^{2}-1$ leaves us with

(98) $$q\left(x,t\right)={\left[-A_{1}\left(\frac{sn\left(\xi \right)dn\left(\xi \right)}{cn\left(\xi \right)}\right)\right]}^{\frac{1}{n+1}}e^{i\left[-kx+\omega t-\sigma^{2}t+\sigma W\left(t\right)+\theta_{0}\right]}.$$

A dark soliton is presented by $m\to 1^{-}$:

(99) $$q\left(x,t\right)={\left[-A_{1}\text{tanh}\left(\xi \right)\right]}^{\frac{1}{n+1}}e^{i\left[-kx+\omega t-\sigma^{2}t+\sigma W\left(t\right)+\theta_{0}\right]}.$$

*Case 3***:** Taking G(ξ)=dn(ξ), $e_{0}=m^{2}-1,e_{2}=-1,e_{1}=2-m^{2}$ allows us

(100) $$q\left(x,t\right)={\left[-m^{2}A_{1}\left(\frac{sn\left(\xi \right)cn\left(\xi \right)}{dn\left(\xi \right)}\right)\right]}^{\frac{1}{n+1}}e^{i\left[-kx+\omega t-\sigma^{2}t+\sigma W\left(t\right)+\theta_{0}\right]}.$$

The dark soliton (99) is constructed by $m\to 1^{-}$.

*Case 4***:** Choosing G(ξ)=ns(ξ) or G(ξ)=dc(ξ), $e_{0}=m^{2},e_{2}=1,e_{1}=-\left(m^{2}+1\right)$ paves way to

(101) $$q\left(x,t\right)={\left[-A_{1}\left(\frac{ds\left(\xi \right)cs\left(\xi \right)}{ns\left(\xi \right)}\right)\right]}^{\frac{1}{n+1}}e^{i\left[-kx+\omega t-\sigma^{2}t+\sigma W\left(t\right)+\theta_{0}\right]},$$

or

(102) $$q\left(x,t\right)={\left[\left(1-m^{2}\right)A_{1}\left(\frac{nc\left(\xi \right)sc\left(\xi \right)}{dc\left(\xi \right)}\right)\right]}^{\frac{1}{n+1}}e^{i\left[-kx+\omega t-\sigma^{2}t+\sigma W\left(t\right)+\theta_{0}\right]}.$$

A straddled soliton is recovered by $m\to 1^{-}$ in Eq. (54):

(103) $$q\left(x,t\right)={\left\{-A_{1}\left[\text{coth}\left(\xi \right)-\text{tanh}\left(\xi \right)\right]\right\}}^{\frac{1}{n+1}}e^{i\left[-kx+\omega t-\sigma^{2}t+\sigma W\left(t\right)+\theta_{0}\right]}.$$

*Case 5***:** Taking G(ξ)=nd(ξ), $e_{0}=-1,e_{2}=m^{2}-1,e_{1}=2-m^{2}$ gives rise to

(104) $$q\left(x,t\right)={\left[m^{2}A_{1}\left(\frac{sd\left(\xi \right)cd\left(\xi \right)}{nd\left(\xi \right)}\right)\right]}^{\frac{1}{n+1}}e^{i\left[-kx+\omega t-\sigma^{2}t+\sigma W\left(t\right)+\theta_{0}\right]}.$$

A dark soliton is structured by $m\to 1^{-}$:

(105) $$q\left(x,t\right)={\left[A_{1}\text{tanh}\left(\xi \right)\right]}^{\frac{1}{n+1}}e^{i\left[-kx+\omega t-\sigma^{2}t+\sigma W\left(t\right)+\theta_{0}\right]}.$$

*Case 6***:** Choosing G(ξ)=sc(ξ), $e_{0}=1,e_{2}=1-m^{2},e_{1}=2-m^{2}$ causes to

(106) $$q\left(x,t\right)={\left[A_{1}\left(\frac{nc\left(\xi \right)dc\left(\xi \right)}{sc\left(\xi \right)}\right)\right]}^{\frac{1}{n+1}}e^{i\left[-kx+\omega t-\sigma^{2}t+\sigma W\left(t\right)+\theta_{0}\right]}.$$

A singular soliton is structured by $m\to 1^{-}$:

(107) $$q\left(x,t\right)={\left[A_{1}\text{coth}\left(\xi \right)\right]}^{\frac{1}{n+1}}e^{i\left[-kx+\omega t-\sigma^{2}t+\sigma W\left(t\right)+\theta_{0}\right]}.$$

*Case 7***:** Taking G(ξ)=cs(ξ), $e_{0}=1-m^{2},e_{2}=1,e_{1}=2-m^{2}$ leads to

(108) $$q\left(x,t\right)={\left[-A_{1}\left(\frac{ns\left(\xi \right)ds\left(\xi \right)}{cs\left(\xi \right)}\right)\right]}^{\frac{1}{n+1}}e^{i\left[-kx+\omega t-\sigma^{2}t+\sigma W\left(t\right)+\theta_{0}\right]}.$$

A singular soliton is recovered by $m\to 1^{-}$:

(109) $$q\left(x,t\right)={\left[-A_{1}\text{coth}\left(\xi \right)\right]}^{\frac{1}{n+1}}e^{i\left[-kx+\omega t-\sigma^{2}t+\sigma W\left(t\right)+\theta_{0}\right]}.$$

*Case 8***:** Choosing G(ξ)=ns(ξ)±cs(ξ), $e_{0}=\frac{1}{4},e_{2}=\frac{1}{4},e_{1}=\frac{1}{2}\left(1-2m^{2}\right)$ gives

(110) $$q\left(x,t\right)={\left[A_{1}ds\left(\xi \right)\right]}^{\frac{1}{n+1}}e^{i\left[-kx+\omega t-\sigma^{2}t+\sigma W\left(t\right)+\theta_{0}\right]}.$$

A singular soliton is constructed by $m\to 1^{-}$:

(111) $$q\left(x,t\right)={\left[A_{1}csch\left(\xi \right)\right]}^{\frac{1}{n+1}}e^{i\left[-kx+\omega t-\sigma^{2}t+\sigma W\left(t\right)+\theta_{0}\right]}.$$

*Case 9***:** Taking G(ξ)=nc(ξ)±sc(ξ), $e_{0}=\frac{1}{4}\left(1-m^{2}\right),e_{2}=\frac{1}{4}\left(1-m^{2}\right),e_{1}=\frac{1}{2}\left(1+m^{2}\right)$ yields

(112) $$q\left(x,t\right)={\left[A_{1}dc\left(\xi \right)\right]}^{\frac{1}{n+1}}e^{i\left[-kx+\omega t-\sigma^{2}t+\sigma W\left(t\right)+\theta_{0}\right]}.$$

*Case 10***:** Choosing G(ξ)=ns(ξ)±ds(ξ), $e_{0}=\frac{m^{2}}{4},e_{2}=\frac{1}{4},e_{1}=\frac{1}{2}\left(m^{2}-2\right)$ leads to

(113) $$q\left(x,t\right)={\left[A_{1}cs\left(\xi \right)\right]}^{\frac{1}{n+1}}e^{i\left[-kx+\omega t-\sigma^{2}t+\sigma W\left(t\right)+\theta_{0}\right]}.$$

Here, the singular soliton (111) is presented by $m\to 1^{-}$.

In the case of Polynomial-law nonlinearity, we arrive at:

(114) $$F\left(|q{|}^{2}\right)=k_{1}\left|q{|}^{2}+k_{2}\right|q{|}^{4}+k_{3}|q{|}^{6},\;k_{3}\neq 0,$$

where $k_{3},k_{2}$ and $k_{1}$ are arbitrary constants. Also, Eq. (1) sticks out as

(115) $$\begin{array}{c} iq_{t}+aq_{xx}+\left(k_{1}\left|q{|}^{2}+k_{2}\right|q{|}^{4}+k_{3}|q{|}^{6}\right)q+\sigma q\frac{dW\left(t\right)}{dt}=\\ \frac{1}{|q{|}^{2}q^{*}}\left\{\alpha |q{|}^{2}{\left(|q{|}^{2}\right)}_{xx}-\beta {\left[{\left(|q{|}^{2}\right)}_{x}\right]}^{2}\right\}+\gamma q+i\left[\lambda_{1}q_{x}+\mu_{1}{\left(|q{|}^{2}q\right)}_{x}+\theta_{1}{\left(|q{|}^{2}\right)}_{x}q+\upsilon_{1}|q{|}^{2}q_{x}\right], \end{array}$$

while Eq. (8) shapes up as

(116) $$\begin{array}{c} \left(a-2\alpha \right)\phi \phi^{\prime \prime }-\left[ak^{2}+\omega -\sigma^{2}+\gamma +\lambda_{1}k\right]\phi^{2}+k_{2}\phi^{6}+k_{3}\phi^{8}\\ +2\left(2\beta -\alpha \right)\phi^{{}^{\prime }2}+\left[k_{1}-k\left(\mu_{1}+\upsilon_{1}\right)\right]\phi^{4}=0. \end{array}$$

Balancing $\phi^{8}$ with $\phi \phi^{\prime \prime }$ in Eq. (116) leaves us with $N=\frac{1}{3}$. Consider the transformation:

(117) $$\phi \left(\xi \right)={\left[\psi \left(\xi \right)\right]}^{\frac{1}{3}}.$$

Substituting (117) into Eq. (116), we get

(118) $$\begin{array}{c} 3\left(a-2\alpha \right)\psi \psi^{\prime \prime }-9\left[a\;k^{2}+\lambda_{1}k-\sigma^{2}+\gamma +\omega \right]\psi^{2}+9\left[k_{1}-\left(\mu_{1}+\upsilon_{1}\right)k\right]\psi^{\frac{8}{3}}\\ +9k_{2}\psi^{\frac{10}{3}}+9k_{3}\psi^{4}-\left(2a-2\alpha -4\beta \right)\psi^{{}^{\prime }2}=0. \end{array}$$

For Eq. (118) to be integrated, we select $k_{1}=\left(\mu_{1}+\upsilon_{1}\right)k$ and $k_{2}=0$. Thus, Eq. (118) simplifies to

(119) $$\begin{array}{c} 3\left(a-2\alpha \right)\psi \psi^{\prime \prime }-9\left[a\;k^{2}+\omega -\sigma^{2}+\lambda_{1}k+\gamma \right]\psi^{2}\\ +9k_{3}\psi^{4}-2\left(a-\alpha -2\beta \right)\psi^{{}^{\prime }2}=0. \end{array}$$

Balancing $\psi^{4}$ with $\psi \psi^{\prime \prime }$ in Eq. (119) leads to N=1. Next Eq. (12) reads as

(120) $$\psi \left(\xi \right)=A_{0}+A_{1}\left(\frac{G^{\prime }}{G}\right),\;A_{1}\neq 0.$$

Substituting (120) along with (13) into Eq. (119) causes to

$A_{0}=0,\;A_{1}=\sqrt{\frac{-\sigma^{2}+\omega +a\;k^{2}+k\lambda_{1}+\gamma }{e_{1}k_{3}}},$along with the constraint conditions

(121) $$\begin{array}{c} \alpha =\frac{3a\;k^{2}+3\lambda_{1}k+2ae_{1}-3\sigma^{2}+3\gamma +3\omega }{4e_{1}},\\ \beta =\frac{-3a\;k^{2}-3\lambda_{1}k+2ae_{1}+3\sigma^{2}-3\gamma -3\omega }{8e_{1}}, \end{array}$$

provided $e_{1}k_{3}\left[-\sigma^{2}+\omega +a\;k^{2}+k\lambda_{1}+\gamma \right]>0$.

Substituting (121) along with (117) and (120) into (2), we get

(122) $$q\left(x,t\right)={\left\{\sqrt{\frac{-\sigma^{2}+\omega +a\;k^{2}+k\lambda_{1}+\gamma }{e_{1}k_{3}}}\left(\frac{G^{\prime }}{G}\right)\right\}}^{\frac{1}{3}}e^{i\left[-kx+\omega t-\sigma^{2}t+\sigma W\left(t\right)+\theta_{0}\right]},$$

provided $A_{1}\left(\frac{G^{\prime }}{G}\right)>0$.

*Case 1***:** Taking G(ξ)=sn(ξ) or G(ξ)=cd(ξ), $e_{0}=1,e_{2}=m^{2},e_{1}=-\left(m^{2}+1\right)$ yields

(123) $$q\left(x,t\right)={\left\{\sqrt{-\frac{-\sigma^{2}+\omega +a\;k^{2}+k\lambda_{1}+\gamma }{\left(m^{2}+1\right)k_{3}}}\;\left[\frac{cn\left(\xi \right)dn\left(\xi \right)}{sn\left(\xi \right)}\right]\right\}}^{\frac{1}{3}}e^{i\left[-kx+\omega t-\sigma^{2}t+\sigma W\left(t\right)+\theta_{0}\right]},$$

or

(124) $$\begin{array}{c} q\left(x,t\right)=\\ {\left\{-\sqrt{-\frac{-\sigma^{2}+\omega +a\;k^{2}+k\lambda_{1}+\gamma }{\left(m^{2}+1\right)k_{3}}}\;\left[\left(1-m^{2}\right)\left(\frac{sd\left(\xi \right)nd\left(\xi \right)}{cd\left(\xi \right)}\right)\right]\right\}}^{\frac{1}{3}}e^{i\left[-kx+\omega t-\sigma^{2}t+\sigma W\left(t\right)+\theta_{0}\right]}. \end{array}$$

A straddled soliton is formulated by $m\to 1^{-}$ in Eq. (123):

(125) $$q\left(x,t\right)={\left\{\sqrt{-\frac{-\sigma^{2}+\omega +a\;k^{2}+k\lambda_{1}+\gamma }{2k_{3}}}\;\left[\text{coth}\left(\xi \right)-\text{tanh}\left(\xi \right)\right]\right\}}^{\frac{1}{3}}e^{i\left[-kx+\omega t-\sigma^{2}t+\sigma W\left(t\right)+\theta_{0}\right]},$$

provided $k_{3}\left[-\sigma^{2}+\omega +a\;k^{2}+\lambda_{1}k+\gamma \right]<0$.

*Case 2***:** Choosing G(ξ)=cn(ξ), $e_{0}=1-m^{2},e_{2}=-m^{2},e_{1}=2m^{2}-1$ gives

(126) $$q\left(x,t\right)={\left\{-\sqrt{\frac{-\sigma^{2}+\omega +a\;k^{2}+k\lambda_{1}+\gamma }{\left(2m^{2}-1\right)k_{3}}}\left[\frac{sn\left(\xi \right)dn\left(\xi \right)}{cn\left(\xi \right)}\right]\right\}}^{\frac{1}{3}}e^{i\left[-kx+\omega t-\sigma^{2}t+\sigma W\left(t\right)+\theta_{0}\right]}$$

provided $\left[-\sigma^{2}+\omega +a\;k^{2}+k\lambda_{1}+\gamma \right]\left(2m^{2}-1\right)k_{3}>0$.

A dark soliton is structured by $m\to 1^{-}$:

(127) $$q\left(x,t\right)={\left\{-\sqrt{\frac{-\sigma^{2}+\omega +a\;k^{2}+k\lambda_{1}+\gamma }{k_{3}}}\;\text{tanh}\left(\xi \right)\right\}}^{\frac{1}{3}}e^{i\left[-kx+\omega t-\sigma^{2}t+\sigma W\left(t\right)+\theta_{0}\right]}$$

provided $\left[-\sigma^{2}+\omega +a\;k^{2}+k\lambda_{1}+\gamma \right]k_{3}>0$.

*Case 3***:** Taking G(ξ)=dn(ξ), $e_{0}=m^{2}-1,e_{2}=-1,e_{1}=2-m^{2}$ leads to

(128) $$q\left(x,t\right)={\left\{-\sqrt{\frac{-\sigma^{2}+\omega +a\;k^{2}+k\lambda_{1}+\gamma }{\left(2-m^{2}\right)k_{3}}}\left[m^{2}\left(\frac{sn\left(\xi \right)cn\left(\xi \right)}{dn\left(\xi \right)}\right)\right]\right\}}^{\frac{1}{3}}e^{i\left[-kx+\omega t-\sigma^{2}t+\sigma W\left(t\right)+\theta_{0}\right]},$$

provided

$\left[-\sigma^{2}+\omega +a\;k^{2}+k\lambda_{1}+\gamma \right]k_{3}>0.$

The dark soliton (127) is recovered by $m\to 1^{-}$.

*Case 4***:** Choosing G(ξ)=ns(ξ) or G(ξ)=dc(ξ), $e_{0}=m^{2},e_{2}=1,e_{1}=-\left(m^{2}+1\right)$ causes to

(129) $$q\left(x,t\right)={\left\{-\sqrt{-\frac{-\sigma^{2}+\omega +a\;k^{2}+k\lambda_{1}+\gamma }{\left(m^{2}+1\right)k_{3}}}\left[\frac{ds\left(\xi \right)cs\left(\xi \right)}{ns\left(\xi \right)}\right]\right\}}^{\frac{1}{3}}e^{i\left[-kx+\omega t-\sigma^{2}t+\sigma W\left(t\right)+\theta_{0}\right]},$$

or

q(x,t)=

(130) $${\left\{\sqrt{-\frac{-\sigma^{2}+\omega +a\;k^{2}+k\lambda_{1}+\gamma }{\left(m^{2}+1\right)k_{3}}}\left[\left(1-m^{2}\right)\left(\frac{nc\left(\xi \right)sc\left(\xi \right)}{dc\left(\xi \right)}\right)\right]\right\}}^{\frac{1}{3}}e^{i\left[-kx+\omega t-\sigma^{2}t+\sigma W\left(t\right)+\theta_{0}\right]}.$$

A straddled soliton is constructed by $m\to 1^{-}$ in Eq. (129):

(131) $$q\left(x,t\right)={\left\{-\sqrt{-\frac{-\sigma^{2}+\omega +a\;k^{2}+k\lambda_{1}+\gamma }{2k_{3}}}\;\left[\text{coth}\left(\xi \right)-\text{tanh}\left(\xi \right)\right]\right\}}^{\frac{1}{3}}e^{i\left[-kx+\omega t-\sigma^{2}t+\sigma W\left(t\right)+\theta_{0}\right]},$$

provided $\left[-\sigma^{2}+\omega +a\;k^{2}+k\lambda_{1}+\gamma \right]k_{3}<0$.

*Case 5***:** Taking G(ξ)=nd(ξ), $e_{0}=-1,e_{2}=m^{2}-1,e_{1}=2-m^{2}$ gives rise to

(132) $$q\left(x,t\right)={\left\{\sqrt{\frac{-\sigma^{2}+\omega +a\;k^{2}+k\lambda_{1}+\gamma }{\left(2-m^{2}\right)k_{3}}}\left[m^{2}\left(\frac{sd\left(\xi \right)cd\left(\xi \right)}{nd\left(\xi \right)}\right)\right]\right\}}^{\frac{1}{3}}e^{i\left[-kx+\omega t-\sigma^{2}t+\sigma W\left(t\right)+\theta_{0}\right]}.$$

A dark soliton is presented by $m\to 1^{-}$:

(133) $$q\left(x,t\right)={\left\{\sqrt{\frac{-\sigma^{2}+\omega +a\;k^{2}+\lambda_{1}k+\gamma }{k_{3}}}\;\text{tanh}\left(\xi \right)\right\}}^{\frac{1}{3}}e^{i\left[-kx+\omega t-\sigma^{2}t+\sigma W\left(t\right)+\theta_{0}\right]},$$

provided $\left[-\sigma^{2}+\omega +a\;k^{2}+k\lambda_{1}+\gamma \right]k_{3}>0$.

*Case 6***:** Choosing G(ξ)=sc(ξ), $e_{0}=1,e_{2}=1-m^{2},e_{1}=2-m^{2}$ paves way to

(134) $$q\left(x,t\right)={\left\{\sqrt{\frac{-\sigma^{2}+\omega +a\;k^{2}+k\lambda_{1}+\gamma }{\left(2-m^{2}\right)k_{3}}}\left[\frac{nc\left(\xi \right)dc\left(\xi \right)}{sc\left(\xi \right)}\right]\right\}}^{\frac{1}{3}}e^{i\left[-kx+\omega t-\sigma^{2}t+\sigma W\left(t\right)+\theta_{0}\right]}.$$

A singular soliton is extracted by $m\to 1^{-}$:

(135) $$q\left(x,t\right)={\left\{\sqrt{\frac{-\sigma^{2}+\omega +a\;k^{2}+\lambda_{1}k+\gamma }{k_{3}}}\;\text{coth}\left(\xi \right)\right\}}^{\frac{1}{3}}e^{i\left[-kx+\omega t-\sigma^{2}t+\sigma W\left(t\right)+\theta_{0}\right]},$$

provided

$\left[-\sigma^{2}+\omega +a\;k^{2}+k\lambda_{1}+\gamma \right]k_{3}>0.$

*Case 7***:** Taking G(ξ)=cs(ξ), $e_{0}=1-m^{2},e_{2}=1,e_{1}=2-m^{2}$ allows us

(136) $$q\left(x,t\right)={\left\{-\sqrt{\frac{-\sigma^{2}+\omega +a\;k^{2}+k\lambda_{1}+\gamma }{\left(2-m^{2}\right)k_{3}}}\left[\frac{ns\left(\xi \right)ds\left(\xi \right)}{cs\left(\xi \right)}\right]\right\}}^{\frac{1}{3}}e^{i\left[-kx+\omega t-\sigma^{2}t+\sigma W\left(t\right)+\theta_{0}\right]}.$$

A singular soliton is modeled by $m\to 1^{-}$:

(137) $$q\left(x,t\right)={\left\{-\sqrt{\frac{-\sigma^{2}+\omega +a\;k^{2}+k\lambda_{1}+\gamma }{k_{3}}}\;\text{coth}\left(\xi \right)\right\}}^{\frac{1}{3}}e^{i\left[-kx+\omega t-\sigma^{2}t+\sigma W\left(t\right)+\theta_{0}\right]},$$

provided $\left[-\sigma^{2}+\omega +a\;k^{2}+k\lambda_{1}+\gamma \right]k_{3}>0$.

*Case 8***:** Choosing G(ξ)=ns(ξ)±cs(ξ), $e_{0}=\frac{1}{4},e_{2}=\frac{1}{4},e_{1}=\frac{1}{2}\left(1-2m^{2}\right)$ leaves us with

(138) $$q\left(x,t\right)={\left\{\sqrt{-\frac{2\left[-\sigma^{2}+\omega +a\;k^{2}+k\lambda_{1}+\gamma \right]}{\left(2m^{2}-1\right)k_{3}}}ds\left(\xi \right)\;\right\}}^{\frac{1}{3}}e^{i\left[-kx+\omega t-\sigma^{2}t+\sigma W\left(t\right)+\theta_{0}\right]},$$

provided

$\left[-\sigma^{2}+\omega +a\;k^{2}+k\lambda_{1}+\gamma \right]\left(2m^{2}-1\right)k_{3}<0.$

A singular soliton is formulated by $m\to 1^{-}$:

(139) $$q\left(x,t\right)={\left\{\sqrt{-\frac{2\left[-\sigma^{2}+\omega +a\;k^{2}+\lambda_{1}k+\gamma \right]}{k_{3}}}\;\text{csch}\left(\xi \right)\right\}}^{\frac{1}{3}}e^{i\left[-kx+\omega t-\sigma^{2}t+\sigma W\left(t\right)+\theta_{0}\right]},$$

provided $\left[-\sigma^{2}+\omega +a\;k^{2}+k\lambda_{1}+\gamma \right]k_{3}<0$.

*Case 9***:** Taking G(ξ)=nc(ξ)±sc(ξ), $e_{0}=\frac{1}{4}\left(1-m^{2}\right),e_{2}=\frac{1}{4}\left(1-m^{2}\right),e_{1}=\frac{1}{2}\left(1+m^{2}\right)$ provides us

(140) $$q\left(x,t\right)={\left\{\sqrt{\frac{2\left[-\sigma^{2}+\omega +a\;k^{2}+\lambda_{1}k+\gamma \right]}{k_{3}\left(m^{2}+1\right)}}dc\left(\xi \right)\;\right\}}^{\frac{1}{3}}e^{i\left[-kx+\omega t-\sigma^{2}t+\sigma W\left(t\right)+\theta_{0}\right]},$$

provided

$\left[-\sigma^{2}+\omega +a\;k^{2}+\lambda_{1}k+\gamma \right]k_{3}>0.$

*Case 10***:** Choosing G(ξ)=ns(ξ)±ds(ξ), $e_{0}=\frac{m^{2}}{4},e_{2}=\frac{1}{4},e_{1}=\frac{1}{2}\left(m^{2}-2\right)$ yields

(141) $$q\left(x,t\right)={\left\{\sqrt{\frac{2\left[-\sigma^{2}+\omega +a\;k^{2}+\lambda_{1}k+\gamma \right]}{k_{3}\left(m^{2}-2\right)}}cs\left(\xi \right)\;\right\}}^{\frac{1}{3}}e^{i\left[-kx+\omega t-\sigma^{2}t+\sigma W\left(t\right)+\theta_{0}\right]},$$

provided

$\left[-\sigma^{2}+\omega +a\;k^{2}+\lambda_{1}k+\gamma \right]k_{3}\left(m^{2}-2\right)>0.$

Here the singular soliton (139) is structured by $m\to 1^{-}$.

In the case of Quadratic-law nonlinearity, we arrive at:

(142) $$F\left(|q{|}^{2}\right)=k_{1}\left|q\right|+k_{2}|q{|}^{2},\;k_{1}\neq 0,$$

where $k_{1}$ and $k_{2}$ are constants. Also, Eq. (1) evolves as

(143) $$\begin{array}{c} iq_{t}+aq_{xx}+\left(k_{1}\left|q\right|+k_{2}|q{|}^{2}\right)q+\sigma q\frac{dW\left(t\right)}{dt}=\frac{1}{|q{|}^{2}q^{*}}\left\{\alpha |q{|}^{2}{\left(|q{|}^{2}\right)}_{xx}-\beta {\left[{\left(|q{|}^{2}\right)}_{x}\right]}^{2}\right\}\\ +\gamma q+i\left[\lambda_{1}q_{x}+\mu_{1}{\left(|q{|}^{2}q\right)}_{x}+\theta_{1}{\left(|q{|}^{2}\right)}_{x}q+\upsilon_{1}|q{|}^{2}q_{x}\right], \end{array}$$

while Eq. (8) turns out to be

(144) $$\begin{array}{c} \left(a-2\alpha \right)\phi \phi^{\prime \prime }-\left[ak^{2}+\omega -\sigma^{2}+\gamma +\lambda_{1}k\right]\phi^{2}+k_{1}\phi^{3}\\ +2\left(2\beta -\alpha \right)\phi^{{}^{\prime }2}+\left[k_{2}-k\left(\mu_{1}+\upsilon_{1}\right)\right]\phi^{4}=0. \end{array}$$

Balancing $\phi^{4}$ with $\phi \phi^{\prime \prime }$ in Eq. (144) leads to N=1. Next, Eq. (12) appears as

(145) $$\phi \left(\xi \right)=A_{0}+A_{1}\left(\frac{G^{\prime }}{G}\right),\;A_{1}\neq 0.$$

Substituting (145) along with (13) into Eq. (144) causes to

$A_{0}=\sqrt{e_{1}}\;A_{1},\;A_{1}=A_{1},$along with the constraint conditions

(146) $$\begin{array}{c} \alpha =-\frac{\omega -\sigma^{2}+a\;k^{2}-4ae_{1}+k\lambda_{1}+\gamma }{8e_{1}},\\ \beta =-\frac{\omega -\sigma^{2}+a\;k^{2}+-4ae_{1}+k\lambda_{1}+\gamma }{16e_{1}},\\ k_{1}=\frac{3a\;k^{2}+3\omega -\sigma^{2}+3\lambda_{1}k+3\gamma }{2\sqrt{e_{1}}\;A_{1}},\\ k_{2}=\frac{2\left(\mu_{1}+\upsilon_{1}\right)A_{1}^{2}e_{1}k+\sigma^{2}-\omega -a\;k^{2}-k\lambda_{1}-\gamma }{2A_{1}^{2}e_{1}}, \end{array}$$

where $A_{1}$ is an arbitrary constant and $e_{1}>0$.

Substituting (146) along with (145) into (2), we get

(147) $$q\left(x,t\right)=A_{1}\left[\sqrt{e_{1}}+\left(\frac{G^{\prime }}{G}\right)\right]e^{i\left[-kx+\omega t-\sigma^{2}t+\sigma W\left(t\right)+\theta_{0}\right]}.$$

*Case 1***:** Taking G(ξ)=cn(ξ), $e_{0}=1-m^{2},e_{2}=-m^{2},e_{1}=2m^{2}-1$ provides us

(148) $$q\left(x,t\right)=A_{1}\left[\sqrt{2m^{2}-1}-\frac{sn\left(\xi \right)dn\left(\xi \right)}{cn\left(\xi \right)}\right]e^{i\left[-kx+\omega t-\sigma^{2}t+\sigma W\left(t\right)+\theta_{0}\right]},$$

provided $2m^{2}-1>0$.

A dark soliton is extracted by $m\to 1^{-}$:

(149) $$q\left(x,t\right)=A_{1}\left[1-\text{tanh}\left(\xi \right)\right]e^{i\left[-kx+\omega t-\sigma^{2}t+\sigma W\left(t\right)+\theta_{0}\right]}.$$

*Case 2***:** Choosing G(ξ)=dn(ξ), $e_{0}=m^{2}-1,e_{2}=-1,e_{1}=2-m^{2}$ leaves us with

(150) $$q\left(x,t\right)=A_{1}\left[\sqrt{2-m^{2}}-m^{2}\left(\frac{sn\left(\xi \right)cn\left(\xi \right)}{dn\left(\xi \right)}\right)\right]e^{i\left[-kx+\omega t-\sigma^{2}t+\sigma W\left(t\right)+\theta_{0}\right]}.$$

Here the dark soliton (149) is presented by $m\to 1^{-}$:

*Case 3***:** Taking G(ξ)=nc(ξ), $e_{0}=-m^{2},e_{2}=1-m^{2},e_{1}=2m^{2}-1$ allows us

(151) $$q\left(x,t\right)=A_{1}\left[\sqrt{2m^{2}-1}+\frac{sc\left(\xi \right)dc\left(\xi \right)}{nc\left(\xi \right)}\right]e^{i\left[-kx+\omega t-\sigma^{2}t+\sigma W\left(t\right)+\theta_{0}\right]},$$

provided $2m^{2}-1>0$.

A dark soliton is constructed by $m\to 1^{-}$:

(152) $$q\left(x,t\right)=A_{1}\left[1+\text{tanh}\left(\xi \right)\right]e^{i\left[-kx+\omega t-\sigma^{2}t+\sigma W\left(t\right)+\theta_{0}\right]}.$$

*Case 4***:** Choosing G(ξ)=nd(ξ), $e_{0}=-1,e_{2}=m^{2}-1,e_{1}=2-m^{2}$ paves way to

(153) $$q\left(x,t\right)=A_{1}\left[\sqrt{2-m^{2}}+m^{2}\left(\frac{sd\left(\xi \right)cd\left(\xi \right)}{nd\left(\xi \right)}\right)\right]e^{i\left[-kx+\omega t-\sigma^{2}t+\sigma W\left(t\right)+\theta_{0}\right]}.$$

Here the dark soliton (152) is recovered by $m\to 1^{-}$.

*Case 5***:** Taking G(ξ)=cs(ξ), $e_{0}=1-m^{2},e_{2}=1,e_{1}=2-m^{2}$ gives rise to

(154) $$q\left(x,t\right)=A_{1}\left[\sqrt{2-m^{2}}-\frac{ns\left(\xi \right)ds\left(\xi \right)}{cs\left(\xi \right)}\right]e^{i\left[-kx+\omega t-\sigma^{2}t+\sigma W\left(t\right)+\theta_{0}\right]}.$$

A singular soliton is structured by $m\to 1^{-}$:

(155) $$q\left(x,t\right)=A_{1}\left[1-\text{coth}\left(\xi \right)\right]e^{i\left[-kx+\omega t-\sigma^{2}t+\sigma W\left(t\right)+\theta_{0}\right]}.$$

*Case 6***:** Choosing G(ξ)=sc(ξ), $e_{0}=1,e_{2}=1-m^{2},e_{1}=2-m^{2}$ causes to

(156) $$q\left(x,t\right)=A_{1}\left[\sqrt{2-m^{2}}+\frac{nc\left(\xi \right)dc\left(\xi \right)}{sc\left(\xi \right)}\right]e^{i\left[-kx+\omega t-\sigma^{2}t+\sigma W\left(t\right)+\theta_{0}\right]}.$$

A singular soliton is formulated by $m\to 1^{-}$:

(157) $$q\left(x,t\right)=A_{1}\left[1+\text{coth}\left(\xi \right)\right]e^{i\left[-kx+\omega t-\sigma^{2}t+\sigma W\left(t\right)+\theta_{0}\right]}.$$

*Case 7***:** Taking G(ξ)=nc(ξ)±sc(ξ), $e_{0}=\frac{1}{4}\left(1-m^{2}\right),e_{2}=\frac{1}{4}\left(1-m^{2}\right),e_{1}=\frac{1}{2}\left(1+m^{2}\right)$ leads to

(158) $$q\left(x,t\right)=A_{1}\left[\sqrt{\frac{1}{2}\left(1+m^{2}\right)}+dc\left(\xi \right)\right]e^{i\left[-kx+\omega t-\sigma^{2}t+\sigma W\left(t\right)+\theta_{0}\right]}.$$

In the case of Anti-Cubic-law nonlinearity, we arrive at:

(159) $$F\left(|q{|}^{2}\right)=\frac{k_{1}}{|q{|}^{4}}+k_{2}\left|q{|}^{2}+k_{3}\right|q{|}^{4},\;k_{1}\neq 0,$$

where $k_{3},k_{2}$ and $k_{1}$ are constants. Also, Eq. (1) becomes

(160) $$\begin{array}{c} iq_{t}+aq_{xx}+\left(\frac{k_{1}}{|q{|}^{4}}+k_{2}\left|q{|}^{2}+k_{3}\right|q{|}^{4}\right)q+\sigma q\frac{dW\left(t\right)}{dt}=\frac{1}{|q{|}^{2}q^{*}}\left\{\alpha |q{|}^{2}{\left(|q{|}^{2}\right)}_{xx}-\beta {\left[{\left(|q{|}^{2}\right)}_{x}\right]}^{2}\right\}\\ +\gamma q+i\left[\lambda_{1}q_{x}+\mu_{1}{\left(|q{|}^{2}q\right)}_{x}+\theta_{1}{\left(|q{|}^{2}\right)}_{x}q+\upsilon_{1}|q{|}^{2}q_{x}\right], \end{array}$$

while Eq. (8) turns into

(161) $$\begin{array}{c} \left(a-2\alpha \right)\phi^{3}\phi^{\prime \prime }-\left[ak^{2}+\omega -\sigma^{2}+\gamma +\lambda_{1}k\right]\phi^{4}+k_{1}+k_{3}\phi^{8}\\ +2\left(2\beta -\alpha \right)\phi^{2}\phi^{{}^{\prime }2}+\left[k_{2}-k\left(\mu_{1}+\upsilon_{1}\right)\right]\phi^{6}=0. \end{array}$$

Balancing $\phi^{8}$ with $\phi^{3}\phi^{\prime \prime }$ in Eq. (161) yields $N=\frac{1}{2}$. Consider the restriction:

(162) $$\phi \left(\xi \right)={\left[\psi \left(\xi \right)\right]}^{\frac{1}{2}}.$$

Substituting (162) into Eq. (161), we get

(163) $$\begin{array}{c} 2\left(a-2\alpha \right)\psi \psi^{\prime \prime }-4\left[a\;k^{2}+\omega -\sigma^{2}+\lambda_{1}k+\gamma \right]\psi^{2}+\left(-a+4\beta \right)\psi^{{}^{\prime }2}+4k_{1}\\ +4\left[k_{2}-\left(\upsilon_{1}+\mu_{1}\right)k\right]\psi^{3}+4k_{3}\psi^{4}=0, \end{array}$$

Balancing $\psi^{4}$ with $\psi \psi^{\prime \prime }$ in Eq. (163) leads to N=1. Then Eq. (12) changes to

(164) $$\psi \left(\xi \right)=A_{0}+A_{1}\left(\frac{G^{\prime }}{G}\right),\;A_{1}\neq 0.$$

Substituting (164) along with (13) into Eq. (163) allows us

$A_{0}=0,\;A_{1}=A_{1},$along with the constraint conditions

(165) $$\begin{array}{c} \alpha =\frac{\left(2k_{3}\;A_{1}^{2}+a\right)e_{1}-a\;k^{2}+\sigma^{2}-\omega -\lambda_{1}k-\gamma }{2e_{1}},\;\;\;\;k_{2}=k\left(\mu_{1}+\upsilon_{1}\right),\\ \beta =\frac{\left(4k_{3}\;A_{1}^{2}+a\right)e_{1}-4a\;k^{2}+4\left(-\omega +\sigma^{2}\right)-4\lambda_{1}k-4\gamma }{4e_{1}},\\ k_{1}=-\frac{\left(4e_{0}e_{2}-e_{1}^{2}\right)A_{1}^{2}\left[a\;k^{2}-k_{3}\;A_{1}^{2}e_{1}+\omega -\sigma^{2}+\lambda_{1}k+\gamma \right]}{e_{1}}, \end{array}$$

where $A_{1}$ is an arbitrary constant.

Substituting (165) along with (162) and (164) into (2), we get

(166) $$q\left(x,t\right)={\left[A_{1}\left(\frac{G^{\prime }}{G}\right)\right]}^{\frac{1}{2}}e^{i\left[-kx+\omega t-\sigma^{2}t+\sigma W\left(t\right)+\theta_{0}\right]},$$

provided

$A_{1}\left(\frac{G^{\prime }}{G}\right)>0.$*Case 1***:** Taking G(ξ)=sn(ξ) or G(ξ)=cd(ξ), $e_{0}=1,e_{2}=m^{2},e_{1}=-\left(m^{2}+1\right)$ provides us

(167) $$q\left(x,t\right)={\left[A_{1}\left(\frac{cn\left(\xi \right)dn\left(\xi \right)}{sn\left(\xi \right)}\right)\right]}^{\frac{1}{2}}e^{i\left[-kx+\omega t-\sigma^{2}t+\sigma W\left(t\right)+\theta_{0}\right]},$$

or

(168) $$q\left(x,t\right)={\left[\left(m^{2}-1\right)A_{1}\left(\frac{sd\left(\xi \right)nd\left(\xi \right)}{cd\left(\xi \right)}\right)\right]}^{\frac{1}{2}}e^{i\left[-kx+\omega t-\sigma^{2}t+\sigma W\left(t\right)+\theta_{0}\right]}.$$

A straddled soliton is formulated by $m\to 1^{-}$ in Eq. (167):

(169) $$q\left(x,t\right)={\left\{A_{1}\left[\text{coth}\left(\xi \right)-\text{tanh}\left(\xi \right)\right]\right\}}^{\frac{1}{2}}e^{i\left[-kx+\omega t-\sigma^{2}t+\sigma W\left(t\right)+\theta_{0}\right]}.$$

*Case 2***:** Choosing G(ξ)=cn(ξ), $e_{0}=1-m^{2},e_{2}=-m^{2},e_{1}=2m^{2}-1$ leaves us with

(170) $$q\left(x,t\right)={\left[-A_{1}\left(\frac{sn\left(\xi \right)dn\left(\xi \right)}{cn\left(\xi \right)}\right)\right]}^{\frac{1}{2}}e^{i\left[-kx+\omega t-\sigma^{2}t+\sigma W\left(t\right)+\theta_{0}\right]}.$$

A dark soliton is structured by $m\to 1^{-}$:

(171) $$q\left(x,t\right)={\left[-A_{1}\text{tanh}\left(\xi \right)\right]}^{\frac{1}{2}}e^{i\left[-kx+\omega t-\sigma^{2}t+\sigma W\left(t\right)+\theta_{0}\right]}.$$

*Case 3***:** Taking G(ξ)=dn(ξ), $e_{0}=m^{2}-1,e_{2}=-1,e_{1}=2-m^{2}$ allows us

(172) $$q\left(x,t\right)={\left[-m^{2}A_{1}\left(\frac{sn\left(\xi \right)cn\left(\xi \right)}{dn\left(\xi \right)}\right)\right]}^{\frac{1}{2}}e^{i\left[-kx+\omega t-\sigma^{2}t+\sigma W\left(t\right)+\theta_{0}\right]}.$$

Here the dark soliton (171) is recovered by $m\to 1^{-}$.

*Case 4***:** Choosing G(ξ)=ns(ξ) or G(ξ)=dc(ξ), $e_{0}=m^{2},e_{2}=1,e_{1}=-\left(m^{2}+1\right)$ paves way to

(173) $$q\left(x,t\right)={\left[-A_{1}\left(\frac{ds\left(\xi \right)cs\left(\xi \right)}{ns\left(\xi \right)}\right)\right]}^{\frac{1}{2}}e^{i\left[-kx+\omega t-\sigma^{2}t+\sigma W\left(t\right)+\theta_{0}\right]},$$

or

(174) $$q\left(x,t\right)={\left[\left(1-m^{2}\right)A_{1}\left(\frac{nc\left(\xi \right)sc\left(\xi \right)}{dc\left(\xi \right)}\right)\right]}^{\frac{1}{2}}e^{i\left[-kx+\omega t-\sigma^{2}t+\sigma W\left(t\right)+\theta_{0}\right]}.$$

A straddled soliton is constructed by $m\to 1^{-}$ in Eq. (173):

(175) $$q\left(x,t\right)={\left\{-A_{1}\left[\text{coth}\left(\xi \right)-\text{tanh}\left(\xi \right)\right]\right\}}^{\frac{1}{2}}e^{i\left[-kx+\omega t-\sigma^{2}t+\sigma W\left(t\right)+\theta_{0}\right]}.$$

*Case 5***:** Taking G(ξ)=nd(ξ), $e_{0}=-1,e_{2}=m^{2}-1,e_{1}=2-m^{2}$ gives rise to

(176) $$q\left(x,t\right)={\left[m^{2}A_{1}\left(\frac{sd\left(\xi \right)cd\left(\xi \right)}{nd\left(\xi \right)}\right)\right]}^{\frac{1}{2}}e^{i\left[-kx+\omega t-\sigma^{2}t+\sigma W\left(t\right)+\theta_{0}\right]}.$$

A dark soliton is presented by $m\to 1^{-}$:

(177) $$q\left(x,t\right)={\left[A_{1}\text{tanh}\left(\xi \right)\right]}^{\frac{1}{2}}e^{i\left[-kx+\omega t-\sigma^{2}t+\sigma W\left(t\right)+\theta_{0}\right]}.$$

*Case 6***:** Choosing G(ξ)=sc(ξ), $e_{0}=1,e_{2}=1-m^{2},e_{1}=2-m^{2}$ causes to

(178) $$q\left(x,t\right)={\left[A_{1}\left(\frac{nc\left(\xi \right)dc\left(\xi \right)}{sc\left(\xi \right)}\right)\right]}^{\frac{1}{2}}e^{i\left[-kx+\omega t-\sigma^{2}t+\sigma W\left(t\right)+\theta_{0}\right]}.$$

A singular soliton is extracted by $m\to 1^{-}$:

(179) $$q\left(x,t\right)={\left[A_{1}\text{coth}\left(\xi \right)\right]}^{\frac{1}{2}}e^{i\left[-kx+\omega t-\sigma^{2}t+\sigma W\left(t\right)+\theta_{0}\right]}.$$

*Case 7***:** Taking G(ξ)=cs(ξ), $e_{0}=1-m^{2},e_{2}=1,e_{1}=2-m^{2}$ leads to

(180) $$q\left(x,t\right)={\left[-A_{1}\left(\frac{ns\left(\xi \right)ds\left(\xi \right)}{cs\left(\xi \right)}\right)\right]}^{\frac{1}{2}}e^{i\left[-kx+\omega t-\sigma^{2}t+\sigma W\left(t\right)+\theta_{0}\right]}.$$

A singular soliton is modeled by $m\to 1^{-}$:

(181) $$q\left(x,t\right)={\left[-A_{1}\text{coth}\left(\xi \right)\right]}^{\frac{1}{2}}e^{i\left[-kx+\omega t-\sigma^{2}t+\sigma W\left(t\right)+\theta_{0}\right]}.$$

*Case 8***:** Choosing G(ξ)=ns(ξ)±cs(ξ), $e_{0}=\frac{1}{4},e_{2}=\frac{1}{4},e_{1}=\frac{1}{2}\left(1-2m^{2}\right)$ gives

(182) $$q\left(x,t\right)={\left[A_{1}ds\left(\xi \right)\right]}^{\frac{1}{2}}e^{i\left[-kx+\omega t-\sigma^{2}t+\sigma W\left(t\right)+\theta_{0}\right]}.$$

A singular soliton is formulated by $m\to 1^{-}$:

(183) $$q\left(x,t\right)={\left[A_{1}csch\left(\xi \right)\right]}^{\frac{1}{2}}e^{i\left[-kx+\omega t-\sigma^{2}t+\sigma W\left(t\right)+\theta_{0}\right]}.$$

*Case 9***:** Taking G(ξ)=nc(ξ)±sc(ξ), $e_{0}=\frac{1}{4}\left(1-m^{2}\right),e_{2}=\frac{1}{4}\left(1-m^{2}\right),e_{1}=\frac{1}{2}\left(1+m^{2}\right)$ yields

(184) $$q\left(x,t\right)={\left[A_{1}dc\left(\xi \right)\right]}^{\frac{1}{2}}e^{i\left[-kx+\omega t-\sigma^{2}t+\sigma W\left(t\right)+\theta_{0}\right]}.$$

*Case 10***:** Choosing G(ξ)=ns(ξ)±ds(ξ), $e_{0}=\frac{m^{2}}{4},e_{2}=\frac{1}{4},e_{1}=\frac{1}{2}\left(m^{2}-2\right)$ provides us

(185) $$q\left(x,t\right)={\left[A_{1}cs\left(\xi \right)\right]}^{\frac{1}{2}}e^{i\left[-kx+\omega t-\sigma^{2}t+\sigma W\left(t\right)+\theta_{0}\right]}.$$

Here the singular soliton (183) is structured by $m\to 1^{-}$.

In the case of Generalized Anti-Cubic-law nonlinearity, we arrive at:

(186) $$F\left(|q{|}^{2}\right)=\frac{k_{1}}{|q{|}^{2\left(n+1\right)}}+k_{2}\left|q{|}^{2n}+k_{3}\right|q{|}^{2\left(n+1\right)},\;k_{1}\neq 0,$$

where $k_{3},k_{2}$ and $k_{1}$ are constants. Eq. (1) appears as

(187) $$\begin{array}{c} iq_{t}+aq_{xx}+\left(\frac{k_{1}}{|q{|}^{2\left(n+1\right)}}+k_{2}\left|q{|}^{2n}+k_{3}\right|q{|}^{2\left(n+1\right)}\right)q+\sigma q\frac{dW\left(t\right)}{dt}=\\ \frac{1}{|q{|}^{2}q^{*}}\left\{\alpha |q{|}^{2}{\left(|q{|}^{2}\right)}_{xx}-\beta {\left[{\left(|q{|}^{2}\right)}_{x}\right]}^{2}\right\}+\gamma q+i\left[\lambda_{1}q_{x}+\mu_{1}{\left(|q{|}^{2}q\right)}_{x}+\theta_{1}{\left(|q{|}^{2}\right)}_{x}q+\upsilon_{1}|q{|}^{2}q_{x}\right], \end{array}$$

while Eq. (12) sticks out as

(188) $$\begin{array}{c} \left(a-2\alpha \right)\phi^{2n+1}\phi^{\prime \prime }-\left[ak^{2}+\omega -\sigma^{2}+\gamma +\lambda_{1}k\right]\phi^{2n+2}+k_{1}+k_{2}\phi^{4n+2}+k_{3}\phi^{4n+4}\\ +2\left(2\beta -\alpha \right)\phi^{2n}\phi^{{}^{\prime }2}-k\left(\mu_{1}+\upsilon_{1}\right)\phi^{2n+4}=0. \end{array}$$

Balancing $\phi^{4n+4}$ with $\phi^{2n+1}\phi^{\prime \prime }$ in Eq. (188) provides us $N=\frac{1}{n+1}$. Consider the transformation:

(189) $$\phi \left(\xi \right)={\left[\psi \left(\xi \right)\right]}^{\frac{1}{n+1}},$$

Substituting (189) into Eq. (188), we get

(190) $$\begin{array}{c} \left(n+1\right)\left(a-2\alpha \right)\psi \psi^{\prime \prime }-{\left(n+1\right)}^{2}\left[a\;k^{2}+\omega -\sigma^{2}+\lambda_{1}k+\gamma \right]\psi^{2}+{\left(n+1\right)}^{2}k_{1}\\ +{\left(n+1\right)}^{2}k_{2}\psi^{\frac{4n+2}{n+1}}+{\left(n+1\right)}^{2}k_{3}\psi^{4}+\left[\left(-a+2\alpha \right)n+4\beta -2\alpha \right]\psi^{{}^{\prime }2}-k{\left(n+1\right)}^{2}\left(\mu_{1}+\upsilon_{1}\right)\psi^{\frac{4+2n}{n+1}}=0. \end{array}$$

For Eq. (190) to be integrated, it requires to select $k_{2}=0$ and $\mu_{1}+\upsilon_{1}=0$, Then Eq. (190) reduces to

(191) $$\begin{array}{c} \left(n+1\right)\left(a-2\alpha \right)\psi \psi^{\prime \prime }-{\left(n+1\right)}^{2}\left[a\;k^{2}+\omega -\sigma^{2}+\lambda_{1}k+\gamma \right]\psi^{2}+{\left(n+1\right)}^{2}k_{1}\\ +{\left(n+1\right)}^{2}k_{3}\psi^{4}+\left[\left(-a+2\alpha \right)n+4\beta -2\alpha \right]\psi^{{}^{\prime }2}=0. \end{array}$$

Balancing $\psi \psi^{\prime \prime }$ and $\psi^{4}$ in Eq. (191) leads to N=1. Then Eq. (12) evolves as

(192) $$\psi \left(\xi \right)=A_{0}+A_{1}\left(\frac{G^{\prime }}{G}\right),\;A_{1}\neq 0.$$

Substituting (192) along with (13) into Eq. (191) provides us

$A_{0}=\sqrt{e_{1}}\;A_{1},\;A_{1}=A_{1},$along with the constraint conditions

(193) $$\begin{array}{c} k_{1}=12A_{1}^{4}e_{0}e_{2}k_{3},\;\;\;\;\beta =\frac{k_{3}\left(n+5\right)\left(n+1\right)A_{1}^{2}+a}{4},\\ \alpha =k_{3}\left(n+1\right)A_{1}^{2}+\frac{a}{2},\;\;\;\;\lambda_{1}=\frac{4A_{1}^{2}e_{1}k_{3}-\left(\omega -\sigma^{2}\right)-a\;k^{2}-\gamma }{k}. \end{array}$$

where $A_{1}$ is an arbitrary constant and $e_{1}>0$.

Substituting (193) along with (189) and (192) into (2), we get

(194) $$q\left(x,t\right)={\left\{A_{1}\left[\sqrt{e_{1}}+\left(\frac{G^{\prime }}{G}\right)\right]\right\}}^{\frac{1}{n+1}}e^{i\left[-kx+\omega t-\sigma^{2}t+\sigma W\left(t\right)+\theta_{0}\right]},$$

provided $A_{1}\left[\sqrt{e_{1}}+\left(\frac{G^{\prime }}{G}\right)\right]>0$.

*Case 1***:** Taking G(ξ)=cn(ξ), $e_{0}=1-m^{2},e_{2}=-m^{2},e_{1}=2m^{2}-1$ yields

(195) $$q\left(x,t\right)={\left\{A_{1}\left[\sqrt{2m^{2}-1}-\frac{sn\left(\xi \right)dn\left(\xi \right)}{cn\left(\xi \right)}\right]\right\}}^{\frac{1}{n+1}}e^{i\left[-kx+\omega t-\sigma^{2}t+\sigma W\left(t\right)+\theta_{0}\right]},$$

provided $2m^{2}-1>0$.

A dark soliton is extracted by $m\to 1^{-}$:

(196) $$q\left(x,t\right)={\left\{A_{1}\left[1-\text{tanh}\left(\xi \right)\right]\right\}}^{\frac{1}{n+1}}e^{i\left[-kx+\omega t-\sigma^{2}t+\sigma W\left(t\right)+\theta_{0}\right]}.$$

*Case 2***:** Choosing G(ξ)=dn(ξ), $e_{0}=m^{2}-1,e_{2}=-1,e_{1}=2-m^{2}$ gives

(197) $$q\left(x,t\right)={\left\{A_{1}\left[\sqrt{2-m^{2}}-m^{2}\left(\frac{sn\left(\xi \right)cn\left(\xi \right)}{dn\left(\xi \right)}\right)\right]\right\}}^{\frac{1}{n+1}}e^{i\left[-kx+\omega t-\sigma^{2}t+\sigma W\left(t\right)+\theta_{0}\right]}.$$

Here the dark soliton (196) is presented by $m\to 1^{-}$.

*Case 3***:** Taking G(ξ)=nc(ξ), $e_{0}=-m^{2},e_{2}=1-m^{2},e_{1}=2m^{2}-1$ leads to

(198) $$q\left(x,t\right)={\left\{A_{1}\left[\sqrt{2m^{2}-1}+\frac{sc\left(\xi \right)dc\left(\xi \right)}{nc\left(\xi \right)}\right]\right\}}^{\frac{1}{n+1}}e^{i\left[-kx+\omega t-\sigma^{2}t+\sigma W\left(t\right)+\theta_{0}\right]},$$

provided $2m^{2}-1>0$.

A dark soliton is constructed by $m\to 1^{-}$:

(199) $$q\left(x,t\right)={\left\{A_{1}\left[1+\text{tanh}\left(\xi \right)\right]\right\}}^{\frac{1}{n+1}}e^{i\left[-kx+\omega t-\sigma^{2}t+\sigma W\left(t\right)+\theta_{0}\right]}.$$

*Case 4***:** Choosing G(ξ)=nd(ξ), $e_{0}=-1,e_{2}=m^{2}-1,e_{1}=2-m^{2}$ causes to

(200) $$q\left(x,t\right)={\left\{A_{1}\left[\sqrt{2-m^{2}}+m^{2}\left(\frac{sd\left(\xi \right)cd\left(\xi \right)}{nd\left(\xi \right)}\right)\right]\right\}}^{\frac{1}{n+1}}e^{i\left[-kx+\omega t-\sigma^{2}t+\sigma W\left(t\right)+\theta_{0}\right]}.$$

Here the dark soliton (199) is recovered by $m\to 1^{-}$.

*Case 5***:** Taking G(ξ)=cs(ξ), $e_{0}=1-m^{2},e_{2}=1,e_{1}=2-m^{2}$ gives rise to

(201) $$q\left(x,t\right)={\left\{A_{1}\left[\sqrt{2-m^{2}}-\frac{ns\left(\xi \right)ds\left(\xi \right)}{cs\left(\xi \right)}\right]\right\}}^{\frac{1}{n+1}}e^{i\left[-kx+\omega t-\sigma^{2}t+\sigma W\left(t\right)+\theta_{0}\right]}.$$

A singular soliton is structured by $m\to 1^{-}$:

(202) $$q\left(x,t\right)={\left\{A_{1}\left[1-\text{coth}\left(\xi \right)\right]\right\}}^{\frac{1}{n+1}}e^{i\left[-kx+\omega t-\sigma^{2}t+\sigma W\left(t\right)+\theta_{0}\right]}.$$

*Case 6***:** Choosing G(ξ)=sc(ξ), $e_{0}=1,e_{2}=1-m^{2},e_{1}=2-m^{2}$ paves way to

(203) $$q\left(x,t\right)={\left\{A_{1}\left[\sqrt{2-m^{2}}+\frac{nc\left(\xi \right)dc\left(\xi \right)}{sc\left(\xi \right)}\right]\right\}}^{\frac{1}{n+1}}e^{i\left[-kx+\omega t-\sigma^{2}t+\sigma W\left(t\right)+\theta_{0}\right]}.$$

A singular soliton is formulated by $m\to 1^{-}$:

(204) $$q\left(x,t\right)={\left\{A_{1}\left[1+\text{coth}\left(\xi \right)\right]\right\}}^{\frac{1}{n+1}}e^{i\left[-kx+\omega t-\sigma^{2}t+\sigma W\left(t\right)+\theta_{0}\right]}.$$

*Case 7***:** Taking G(ξ)=nc(ξ)±sc(ξ), $e_{0}=\frac{1}{4}\left(1-m^{2}\right),e_{2}=\frac{1}{4}\left(1-m^{2}\right),e_{1}=\frac{1}{2}\left(1+m^{2}\right)$ allows us

(205) $$q\left(x,t\right)={\left\{A_{1}\left[\sqrt{\frac{1}{2}\left(1+m^{2}\right)}+dc\left(\xi \right)\right]\right\}}^{\frac{1}{n+1}}e^{i\left[-kx+\omega t-\sigma^{2}t+\sigma W\left(t\right)+\theta_{0}\right]}.$$

In the case of Cubic-Quintic-Septic-Nonic-law nonlinearity, we arrive at:

(206) $$F\left(|q{|}^{2}\right)=k_{1}\left|q{|}^{2}+k_{2}\right|q{|}^{4}+k_{3}\left|q{|}^{6}+k_{4}\right|q{|}^{8},\;k_{4}\neq 0,$$

where $k_{j}\left(j=1-4\right)$ are constants. Also, Eq. (1) reads as:

(207) $$\begin{array}{c} iq_{t}+aq_{xx}+\left(k_{1}\left|q{|}^{2}+k_{2}\right|q{|}^{4}+k_{3}\left|q{|}^{6}+k_{4}\right|q{|}^{8}\right)q+\sigma q\frac{dW\left(t\right)}{dt}=\\ \frac{1}{|q{|}^{2}q^{*}}\left\{\alpha |q{|}^{2}{\left(|q{|}^{2}\right)}_{xx}-\beta {\left[{\left(|q{|}^{2}\right)}_{x}\right]}^{2}\right\}+\gamma q+i\left[\lambda_{1}q_{x}+\mu_{1}{\left(|q{|}^{2}q\right)}_{x}+\theta_{1}{\left(|q{|}^{2}\right)}_{x}q+\upsilon_{1}|q{|}^{2}q_{x}\right], \end{array}$$

while Eq. (8) stands as

(208) $$\begin{array}{c} \left(a-2\alpha \right)\phi \phi^{\prime \prime }-\left[ak^{2}+\omega -\sigma^{2}+\gamma +\lambda_{1}k\right]\phi^{2}+k_{2}\phi^{6}+k_{3}\phi^{8}+k_{4}\phi^{10}\\ +2\left(2\beta -\alpha \right)\phi^{{}^{\prime }2}+\left[k_{1}-k\left(\mu_{1}+\upsilon_{1}\right)\right]\phi^{4}=0. \end{array}$$

Balancing $\phi^{10}$ with $\phi \phi^{\prime \prime }$ in Eq. (208) gives $N=\frac{1}{4}$. Consider the transformation:

(209) $$\phi \left(\xi \right)={\left[\psi \left(\xi \right)\right]}^{\frac{1}{4}},$$

Substituting (209) into Eq. (208), we get

(210) $$\begin{array}{c} 4\left(a-2\alpha \right)\psi \psi^{\prime \prime }-16\left[a\;k^{2}+\omega -\sigma^{2}+\lambda_{1}k+\gamma \right]\psi^{2}+16k_{2}\psi^{3}+16k_{3}\psi^{\frac{7}{2}}\\ +16k_{4}\psi^{4}+\left(-3a+4\alpha +4\beta \right)\psi^{{}^{\prime }2}+16\left[k_{1}-\left(\mu_{1}+\upsilon_{1}\right)k\right]\psi^{\frac{5}{2}}=0, \end{array}$$

For Eq. (210) to be integrated, we select $k_{1}=\left(\mu_{1}+\upsilon_{1}\right)k$ and $k_{3}=0$. Thus, Eq. (210) simplifies to

(211) $$\begin{array}{c} 4\left(a-2\alpha \right)\psi \psi^{\prime \prime }-16\left[a\;k^{2}+\omega -\sigma^{2}+\lambda_{1}k+\gamma \right]\psi^{2}+16k_{2}\psi^{3}\\ +16k_{4}\psi^{4}+\left(-3a+4\alpha +4\beta \right)\psi^{{}^{\prime }2}=0. \end{array}$$

Balancing $\psi \psi^{\prime \prime }$ and $\psi^{4}$ in Eq. (211) leads to N=1. Then Eq. (12) comes out as:

(212) $$\psi \left(\xi \right)=A_{0}+A_{1}\left(\frac{G^{\prime }}{G}\right),\;A_{1}\neq 0.$$

Substituting (212) along with (13) into Eq. (211) provides us

$A_{0}=\sqrt{e_{1}}\;A_{1},\;A_{1}=A_{1},$ along with the constraint conditions

(213) $$\begin{array}{c} k_{2}=\frac{-3\left(\sigma^{2}-\omega \right)+3a\;k^{2}+3k\lambda_{1}+3\gamma }{2\sqrt{e_{1}}\;A_{1}},\;\alpha =-\frac{-\left(\sigma^{2}-\omega \right)+a\;k^{2}-ae_{1}+k\lambda_{1}+\gamma }{2e_{1}},\\ k_{4}=-\frac{-\left(\sigma^{2}-\omega \right)+a\;k^{2}+k\lambda_{1}+\gamma }{2A_{1}^{2}e_{1}},\;\beta =\frac{-2\left(\sigma^{2}-\omega \right)+2a\;k^{2}+ae_{1}+2k\lambda_{1}+2\gamma }{4e_{1}}, \end{array}$$

where $A_{1}$ is an arbitrary constant and $e_{1}>0$.

Substituting (213) along with (209) and (212) into (2), we get

(214) $$q\left(x,t\right)={\left\{A_{1}\left[\sqrt{e_{1}}+\left(\frac{G^{\prime }}{G}\right)\right]\right\}}^{\frac{1}{4}}e^{i\left[-kx+\omega t-\sigma^{2}t+\sigma W\left(t\right)+\theta_{0}\right]},$$

provided $A_{1}\left[\sqrt{e_{1}}+\left(\frac{G^{\prime }}{G}\right)\right]>0$.

*Case 1***:** Taking G(ξ)=cn(ξ), $e_{0}=1-m^{2},e_{2}=-m^{2},e_{1}=2m^{2}-1$ yields

(215) $$q\left(x,t\right)={\left\{A_{1}\left[\sqrt{2m^{2}-1}-\frac{sn\left(\xi \right)dn\left(\xi \right)}{cn\left(\xi \right)}\right]\right\}}^{\frac{1}{4}}e^{i\left[-kx+\omega t-\sigma^{2}t+\sigma W\left(t\right)+\theta_{0}\right]},$$

provided $2m^{2}-1>0$.

A dark soliton is modeled by $m\to 1^{-}$:

(216) $$q\left(x,t\right)={\left\{A_{1}\left[1-\text{tanh}\left(\xi \right)\right]\right\}}^{\frac{1}{4}}e^{i\left[-kx+\omega t-\sigma^{2}t+\sigma W\left(t\right)+\theta_{0}\right]}.$$

*Case 2***:** Choosing G(ξ)=dn(ξ), $e_{0}=m^{2}-1,e_{2}=-1,e_{1}=2-m^{2}$ gives

(217) $$q\left(x,t\right)={\left\{A_{1}\left[\sqrt{2-m^{2}}-m^{2}\left(\frac{sn\left(\xi \right)cn\left(\xi \right)}{dn\left(\xi \right)}\right)\right]\right\}}^{\frac{1}{4}}e^{i\left[-kx+\omega t-\sigma^{2}t+\sigma W\left(t\right)+\theta_{0}\right]}.$$

Here the dark soliton (216) is formulated by $m\to 1^{-}$.

*Case 3***:** Taking G(ξ)=nc(ξ), $e_{0}=-m^{2},e_{2}=1-m^{2},e_{1}=2m^{2}-1$ leads to

(218) $$q\left(x,t\right)={\left\{A_{1}\left[\sqrt{2m^{2}-1}+\frac{sc\left(\xi \right)dc\left(\xi \right)}{nc\left(\xi \right)}\right]\right\}}^{\frac{1}{4}}e^{i\left[-kx+\omega t-\sigma^{2}t+\sigma W\left(t\right)+\theta_{0}\right]},$$

provided $2m^{2}-1>0$.

A dark soliton is structured by $m\to 1^{-}$:

(219) $$q\left(x,t\right)={\left\{A_{1}\left[1+\text{tanh}\left(\xi \right)\right]\right\}}^{\frac{1}{4}}e^{i\left[-kx+\omega t-\sigma^{2}t+\sigma W\left(t\right)+\theta_{0}\right]}.$$

*Case 4***:** Choosing G(ξ)=nd(ξ), $e_{0}=-1,e_{2}=m^{2}-1,e_{1}=2-m^{2}$ causes to

(220) $$q\left(x,t\right)={\left\{A_{1}\left[\sqrt{2-m^{2}}+m^{2}\left(\frac{sd\left(\xi \right)cd\left(\xi \right)}{nd\left(\xi \right)}\right)\right]\right\}}^{\frac{1}{4}}e^{i\left[-kx+\omega t-\sigma^{2}t+\sigma W\left(t\right)+\theta_{0}\right]}.$$

Here the dark soliton (219) is recovered by $m\to 1^{-}$.

*Case 5***:** Taking G(ξ)=cs(ξ), $e_{0}=1-m^{2},e_{2}=1,e_{1}=2-m^{2}$ gives rise to

(221) $$q\left(x,t\right)={\left\{A_{1}\left[\sqrt{2-m^{2}}-\frac{ns\left(\xi \right)ds\left(\xi \right)}{cs\left(\xi \right)}\right]\right\}}^{\frac{1}{4}}e^{i\left[-kx+\omega t-\sigma^{2}t+\sigma W\left(t\right)+\theta_{0}\right]}.$$

A singular soliton is constructed by $m\to 1^{-}$:

(222) $$q\left(x,t\right)={\left\{A_{1}\left[1-\text{coth}\left(\xi \right)\right]\right\}}^{\frac{1}{4}}e^{i\left[-kx+\omega t-\sigma^{2}t+\sigma W\left(t\right)+\theta_{0}\right]}.$$

*Case 6***:** Choosing G(ξ)=sc(ξ), $e_{0}=1,e_{2}=1-m^{2},e_{1}=2-m^{2}$ paves way to

(223) $$q\left(x,t\right)={\left\{A_{1}\left[\sqrt{2-m^{2}}+\frac{nc\left(\xi \right)dc\left(\xi \right)}{sc\left(\xi \right)}\right]\right\}}^{\frac{1}{4}}e^{i\left[-kx+\omega t-\sigma^{2}t+\sigma W\left(t\right)+\theta_{0}\right]}.$$

A singular soliton is presented by $m\to 1^{-}$:

(224) $$q\left(x,t\right)={\left\{A_{1}\left[1+\text{coth}\left(\xi \right)\right]\right\}}^{\frac{1}{4}}e^{i\left[-kx+\omega t-\sigma^{2}t+\sigma W\left(t\right)+\theta_{0}\right]}.$$

*Case 7***:** Taking G(ξ)=nc(ξ)±sc(ξ), $e_{0}=\frac{1}{4}\left(1-m^{2}\right),e_{2}=\frac{1}{4}\left(1-m^{2}\right),e_{1}=\frac{1}{2}\left(1+m^{2}\right)$ allows us

(225) $$q\left(x,t\right)={\left\{A_{1}\left[\sqrt{\frac{1}{2}\left(1+m^{2}\right)}+dc\left(\xi \right)\right]\right\}}^{\frac{1}{n+1}}e^{i\left[-kx+\omega t-\sigma^{2}t+\sigma W\left(t\right)+\theta_{0}\right]}.$$

## Method validation

**Fig. 1:**
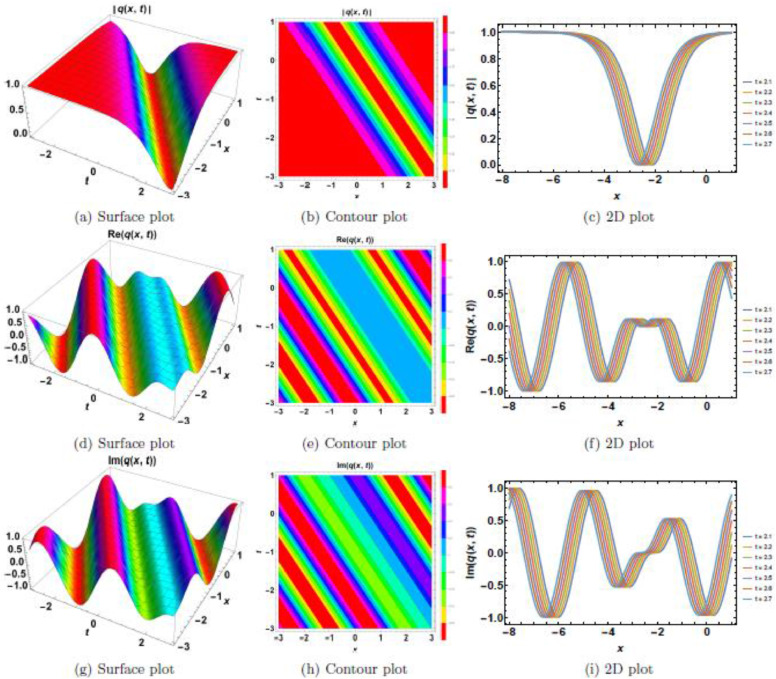
Dark soliton’s waveform given σ=0.

**Fig. 2:**
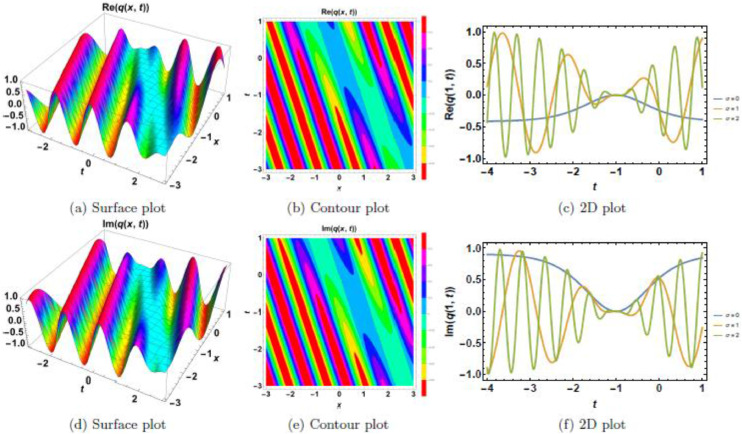
Dark soliton’s waveform given σ=2.

**Fig. 3:**
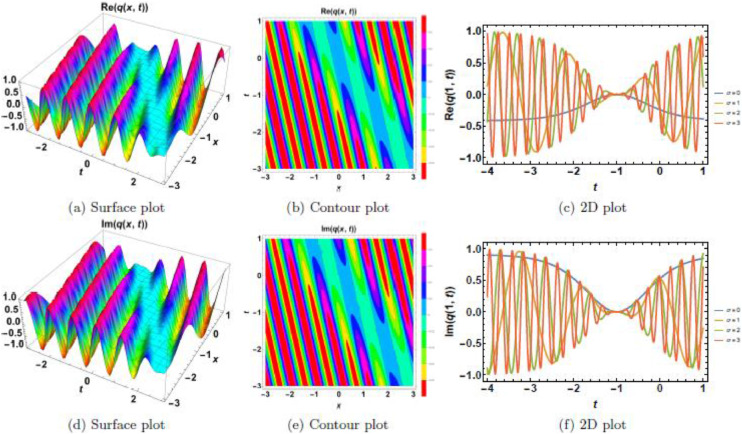
Dark soliton’s waveform given σ=3.

**Fig. 4:**
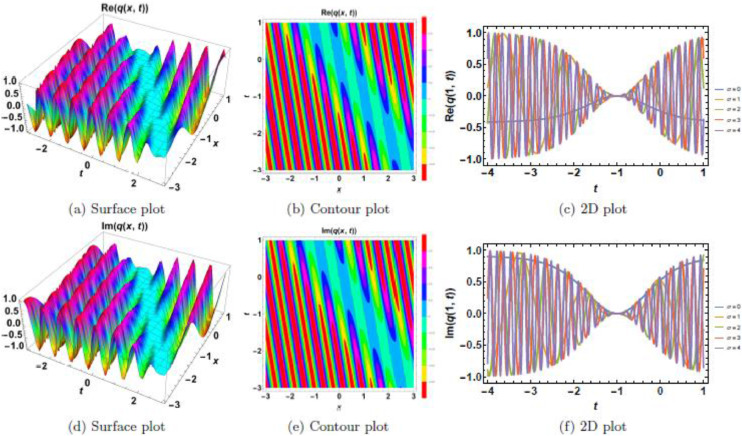
Dark soliton’s waveform given σ=4.

**Fig. 5:**
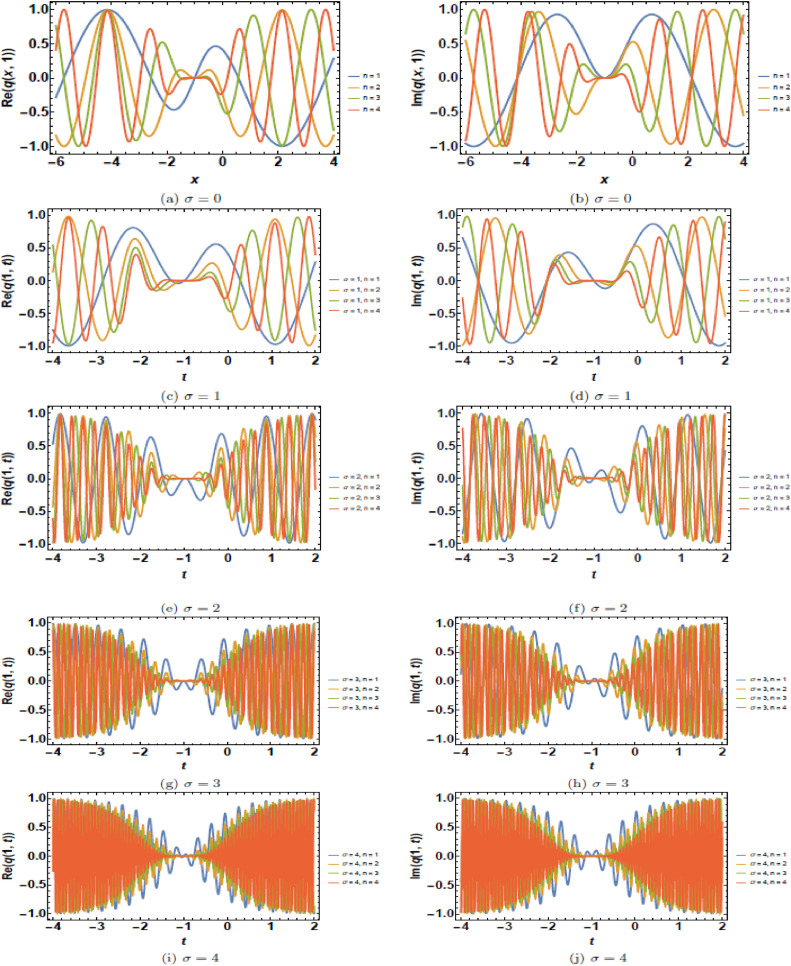
Dark soliton’s waveform given σ=4 and *n* = 4.

**Fig. 6:**
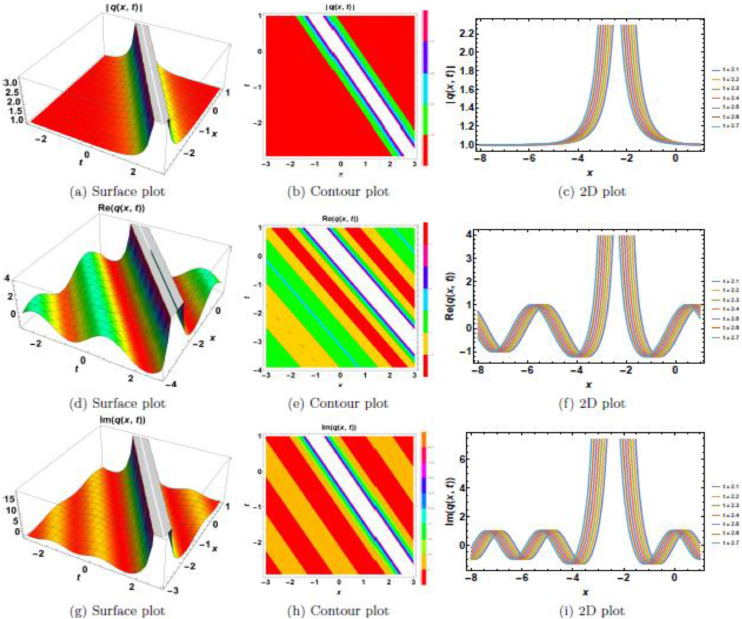
Singular soliton’s waveform given σ=0.

**Fig. 7:**
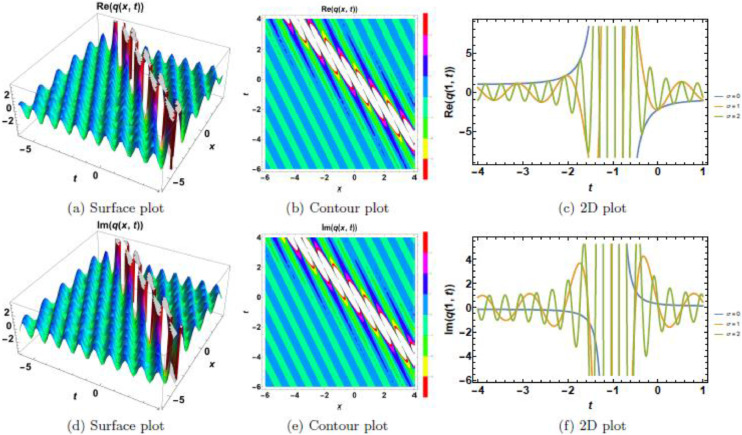
Singular soliton’s waveform given σ=2.

**Fig. 8:**
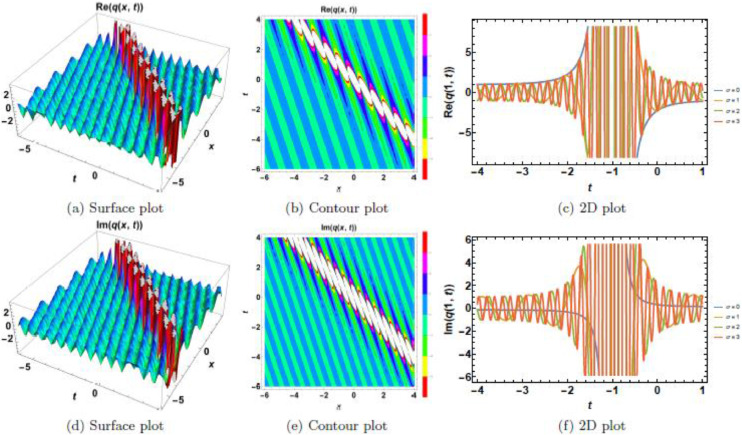
Singular soliton’s waveform given σ=3.

**Fig. 9:**
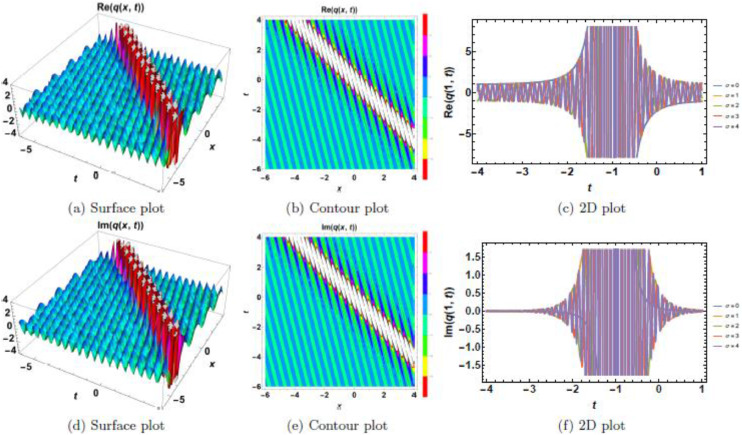
Singular soliton’s waveform given σ=4.

**Fig. 10:**
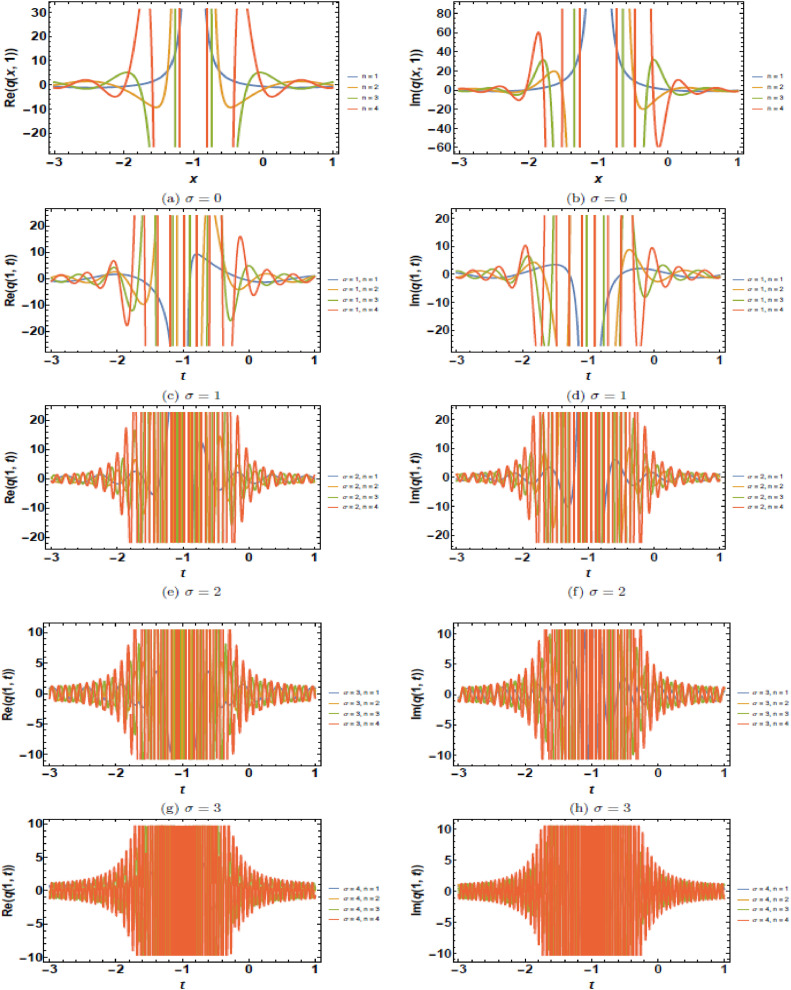
Singular soliton’s waveform given σ=4 and *n* = 4.

This section presents a comprehensive analysis of the obtained optical dark soliton (58) and singular soliton (60), focusing on their structural characteristics under the influence of key physical parameters: $A_{1}=1$, a=1, k=1, λ=1, ω=1, and $\theta_{0}=1$. Specifically, the impact of multiplicative white noise (σ) and power nonlinearity (n) on the soliton dynamics is systematically examined. The analysis is supported by a series of Fig. 1, Fig. 2, Fig. 3, Fig. 4, Fig. 5, Fig. 6, Fig. 7, Fig. 8, Fig. 9, Fig. 10, which illustrate the modulus, real, and imaginary components of the solitons through surface plots, contour plots, and two-dimensional (2D) cross-sectional views. The results provide insights into soliton stability and deformation patterns under varying physical conditions.

The behavior of dark solitons is investigated under different parametric conditions. Fig. 1, Fig. 2, Fig. 3, Fig. 4, Fig. 5 provide a detailed examination of how increasing levels of multiplicative white noise and variations in power nonlinearity influence the soliton structure. Fig. 1 serves as a reference case, presenting the dark soliton in the absence of multiplicative white noise (σ=0). The soliton maintains a well-defined shape with a central intensity dip, a characteristic feature of dark solitons. Surface plots (Figs. 1(a), 1(d), and 1(g)) illustrate the three-dimensional soliton profile. The modulus (Fig. 1(a)) displays a distinct intensity trough, while the real (Fig. 1(d)) and imaginary (Fig. 1(g)) components exhibit symmetric wave-like structures. Contour plots (Figs. 1(b), 1(e), and 1(h)) confirm the stable nature of the soliton, with a well-maintained trough in the modulus. 2D cross-sectional plots (Figs. 1(c), 1(f), and 1(i)) demonstrate that the soliton retains its shape over time, validating its robustness in a noise-free regime. Fig. 2, Fig. 3, Fig. 4 illustrate the soliton response under increasing levels of multiplicative white noise (σ=2,3,4). At σ=2 (Fig. 2), the soliton structure remains largely intact, though minor perturbations appear in the modulus (Fig. 2(a)) and real component (Fig. 2(d)). Contour plots (Figs. 2(b) and 2(e)) indicate the emergence of noise-induced irregularities, while 2D cross-sectional plots (Figs. 2(c) and 2(f)) depict slight oscillations. For σ=3 (Fig. 3), the soliton undergoes more pronounced deformations. The central intensity trough begins to widen (Fig. 3(a)), suggesting an increase in energy dispersion. Contour plots (Figs. 3(b) and 3(e)) show phase irregularities, and 2D plots (Figs. 3(c) and 3(f)) reveal oscillatory distortions. At σ=4 (Fig. 4), significant broadening and deformations become evident. Surface plots (Figs. 4(a) and 4(d)) show a loss of sharpness in the soliton profile, while contour plots (Figs. 4(b) and 4(e)) indicate growing instabilities. The 2D cross-sectional plots (Figs. 4(c) and 4(f)) confirm an increasing degree of structural instability. Fig. 5 investigates the combined effects of power nonlinearity (n=1,2,3,4) and varying white noise levels (σ=1,2,3,4). For lower power nonlinearity (n=1), the soliton exhibits moderate sensitivity to noise, with observable distortions. For higher nonlinearity values (n=4), the soliton retains its structure more effectively, demonstrating enhanced resilience to noise-induced perturbations. A comparative analysis of Figs. 5(a)–5(j) suggests that increasing nonlinearity counteracts dispersive effects, helping maintain soliton integrity.

Singular solitons, characterized by sharp intensity peaks, exhibit a different response to noise and nonlinearity compared to dark solitons. Fig. 6, Fig. 7, Fig. 8, Fig. 9, Fig. 10 analyze these solitons under varying parametric conditions. Fig. 6 presents the fundamental singular soliton profile in the absence of multiplicative white noise. Surface plots (Figs. 6(a), 6(d), and 6(g)) confirm the presence of an intense localized peak. Contour plots (Figs. 6(b), 6(e), and 6(h)) depict a highly confined singularity, reinforcing the sharp nature of the soliton. 2D cross-sectional plots (Figs. 6(c), 6(f), and 6(i)) indicate that the singular soliton maintains a near-singular structure over time. Fig. 7, Fig. 8, Fig. 9 demonstrate how increasing levels of multiplicative white noise affect singular solitons. At σ=2 (Fig. 7), the singularity begins to broaden slightly. Surface plots (Figs. 7(a) and 7(d)) show minimal spreading, while contour plots (Figs. 7(b) and 7(e)) highlight initial oscillatory distortions. For σ=3 (Fig. 8), the soliton undergoes significant perturbations. The sharp intensity peak starts to dissipate, as seen in Figs. 8(a) and 8(d). Contour plots (Figs. 8(b) and 8(e)) reveal phase irregularities, and 2D plots (Figs. 8(c) and 8(f)) depict asymmetrical distortions. At σ=4 (Fig. 9), the singular soliton experiences substantial deformations. Surface plots (Figs. 9(a) and 9(d)) indicate the loss of coherence, while contour plots (Figs. 9(b) and 9(e)) suggest an increasingly chaotic wave profile. Fig. 10 explores the combined effects of power nonlinearity and noise on singular solitons. For low power nonlinearity (n=1), noise-induced distortions are highly pronounced, leading to substantial deformations. At high power nonlinearity (n=4), the soliton exhibits greater structural stability, counteracting the effects of multiplicative white noise. The comparative analysis of Figs. 10(a)–10(j) confirms that increasing nonlinearity enhances soliton robustness, reducing noise-induced perturbations.

The following key observations can be drawn from the results: Dark solitons exhibit greater resilience to noise compared to singular solitons, maintaining structural integrity over a broader range of σ values. Singular solitons are more sensitive to noise, undergoing significant distortions even at moderate levels of σ. Increasing power nonlinearity (n) enhances soliton stability, counteracting the destabilizing effects of multiplicative white noise. For both soliton types, higher σ values lead to more pronounced structural deformations, though the impact is less severe for dark solitons. These findings contribute to a deeper understanding of soliton stability in nonlinear optical systems, with potential implications for optical fiber communication, signal processing, and wave propagation in complex media. Future work could extend this analysis to investigate additional perturbation effects and soliton interactions in higher-dimensional nonlinear models.

This research paper focuses on obtaining optical soliton solutions for the CGLE under the influence of white noise. The study specifically examines the CGLE model in the presence of nine different forms of SPM structures, each of which plays a crucial role in determining the behavior and characteristics of the soliton solutions. These SPM structures affect the phase and amplitude of the propagating optical solitons, making them an integral component of nonlinear fiber optics and optical communication systems.

To achieve the soliton solutions for the CGLE, this work employs the extended $G^{\prime }/G$-expansion scheme as the primary integration technique. This method is a widely recognized analytical approach in nonlinear wave theory, known for its ability to construct various types of soliton solutions, including periodic and singular wave solutions. The core idea of the scheme involves transforming the nonlinear partial differential equation into an ordinary differential equation and then solving it systematically using an auxiliary equation.

For each of the nine considered SPM structures, the soliton solutions were derived through the JEFs. These functions serve as an intermediary mathematical framework that facilitates the extraction of soliton solutions. They provide a bridge between periodic wave solutions and solitons, as they can smoothly transition from one form to another depending on their elliptic modulus parameter, denoted as m. When the elliptic modulus m approaches zero, the JEFs reduce to trigonometric functions, while for m→1, they transform into hyperbolic functions. This property allows soliton solutions to emerge naturally in these two limiting cases, meaning that the methodology successfully retrieves soliton wave structures in these conditions.

The approach is successful in obtaining soliton solutions; however, it is limited by its failure to yield bright optical solitons, which play a central role in nonlinear optics. Given this limitation, the paper emphasizes the need for additional integration methodologies in future studies [[36], [37], [38], [39], [40], [41], [42], [43], [44], [45], [46]]. Researchers must explore alternative mathematical techniques that are capable of retrieving bright optical solitons. Potential strategies may involve using Hirota’s bilinear method [36] simplified Hirota’s method [37], distinct ansatz techniques [38], exponential expansion method [39], WTC–Kruskal method [40], Hirota method [41], Bäcklund transformation [42], binary Darboux transformation [43], generalized Laurent series [44], Darboux dressing transformation [45], generalized Darboux transformation method [46], improved perturbative approaches, variational methods, or different ansatz-based solution techniques that could extend the applicability of the CGLE soliton solutions. These new methodologies must not only be effective but also align with existing soliton solution frameworks to ensure consistency and coherence in nonlinear wave analysis.

Efforts are currently underway to develop and test such alternative integration methods. The results from these ongoing studies, once fully established, will be made publicly available through academic publications. When these new findings are integrated with existing approaches and algorithms, they are expected to enhance the analytical toolkit available for handling the CGLE, ultimately leading to a more comprehensive understanding of soliton dynamics in nonlinear optical systems.

## Limitations

Despite the success in obtaining soliton solutions, this approach has a notable limitation—it fails to generate bright optical soliton solutions, which are fundamental in nonlinear optics. Bright solitons are formed when the balance between nonlinearity and dispersion results in a localized pulse of light with a peak intensity higher than the background. They are particularly important in Quantum Optics, where they are used in applications such as quantum communication, optical signal processing, and secure data transmission. The absence of bright soliton solutions is a significant drawback, as these solutions are crucial for practical implementations in fiber optic technologies and photonics.

## CRediT authorship contribution statement

**Elsayed M.E. Zayed:** Conceptualization, Supervision, Writing – review & editing. **Basel M.M. Saad:** Methodology, Software, Formal analysis. **Ahmed H. Arnous:** Validation, Writing – review & editing. **Yakup Yildirim:** Visualization, Data curation, Investigation, Writing – review & editing. **Ibrahim Zeghaiton Chaloob:** Resources, Writing – review & editing. **Ahmed Shaker Mahmood:** Formal analysis, Writing – review & editing. **Luminita Moraru:** Project administration, Writing – review & editing. **Hamlet Isakhanli:** Supervision, Writing – review & editing. **Anjan Biswas:** Conceptualization, Methodology, Writing – review & editing, Funding acquisition.

## Declaration of competing interest

The authors declare that they have no known competing financial interests or personal relationships that could have appeared to influence the work reported in this paper.

## Data Availability

No data was used for the research described in the article.
